# Disentangling Responses of the Subsurface Microbiome to Wetland Status and Implications for Indicating Ecosystem Functions

**DOI:** 10.3390/microorganisms9020211

**Published:** 2021-01-20

**Authors:** Jie Gao, Miao Liu, Sixue Shi, Ying Liu, Yu Duan, Xianguo Lv, Tsing Bohu, Yuehui Li, Yuanman Hu, Na Wang, Qiuying Wang, Guoqiang Zhuang, Xuliang Zhuang

**Affiliations:** 1 CAS Key Laboratory of Environmental Biotechnology, Research Center for Eco-Environmental Sciences, Chinese Academy of Sciences, Beijing 100085, China; jiegao@rcees.ac.cn (J.G.); lypoppy@mail.ustc.edu.cn (Y.L.); nawang_st@rcees.ac.cn (N.W.); qywang_st@rcees.ac.cn (Q.W.); 2 College of Resources and Environment, University of Chinese Academy of Sciences, Beijing 100049, China; ssx_ssx@126.com; 3 CAS Key Laboratory of Forest Ecology and Management, Institute of Applied Ecology, Chinese Academy of Sciences, Shenyang 110016, China; lium@iae.ac.cn (M.L.); liyh@iae.ac.cn (Y.L.); huym@iae.ac.cn (Y.H.); 4 Beijing Business Department, Beijing Enterprises Water Group Limited, Beijing 100124, China; duanyu@bewg.net.cn; 5 Key Laboratory of Wetland Ecology and Environment, Northeast Institute of Geography and Agroecology, Chinese Academy of Sciences, Changchun 130102, China; luxg@neigae.ac.cn; 6 CSIRO Mineral Resources, 26 Dick Perry Avenue, Kensington, WA 6151, Australia; Qing.Hu@csiro.au

**Keywords:** wetland degradation, microbial community, biogeochemical process, GeoChip arrays, wetland restoration

## Abstract

In this study, we analyzed microbial community composition and the functional capacities of degraded sites and restored/natural sites in two typical wetlands of Northeast China—the Phragmites marsh and the Carex marsh, respectively. The degradation of these wetlands, caused by grazing or land drainage for irrigation, alters microbial community components and functional structures, in addition to changing the aboveground vegetation and soil geochemical properties. Bacterial and fungal diversity at the degraded sites were significantly lower than those at restored/natural sites, indicating that soil microbial groups were sensitive to disturbances in wetland ecosystems. Further, a combined analysis using high-throughput sequencing and GeoChip arrays showed that the abundance of carbon fixation and degradation, and ~95% genes involved in nitrogen cycling were increased in abundance at grazed Phragmites sites, likely due to the stimulating impact of urine and dung deposition. In contrast, the abundance of genes involved in methane cycling was significantly increased in restored wetlands. Particularly, we found that microbial composition and activity gradually shifts according to the hierarchical marsh sites. Altogether, this study demonstrated that microbial communities as a whole could respond to wetland changes and revealed the functional potential of microbes in regulating biogeochemical cycles.

## 1. Introduction

Wetlands, as an important factor of ecosystems, play a critical role in regulating climate change as a carbon sink and a carbon source, provide a unique habitat, and support biodiversity [1]. Despite being the third major ecosystem on Earth, wetlands are threatened by changes in climate and land use [2,3,4]. Land use change is considered the main cause of the loss of wetland ecosystems [5,6]. During the past decades, wetlands have been exploited or used as reservoirs, and as fish and shrimp ponds, whereas others have been used for grazing or have been drained and converted to agricultural, district, residential, and industrial land [7,8]. In Northeast China, agricultural reclamation, cultivation, and grazing are common practice in wetland regions, as the soil nutrients, and extensive vegetation are rich resources, resulting in a reduction of the natural wetland area [9]. The Momoge wetlands of the Songnen Plain are typical wetlands that are severely affected by intensive human activity [10]. However, a series of conservation and restoration policies for wetlands have been implemented in China in recent years, which have played an important role in reconstructing the health of wetland ecosystems [11].

Wetlands are a critical habitat for flora, and the functional/ecological traits of wetland plants are often exerted along environmental gradients. The main plants in the saline-alkaline wetland of Momoge are dominated by *Carex*, *Phragmites*, *Typha*, *Suaeda*, and *Deyeuxia purpurea* [10]. The freshwater marshes in the Sanjiang Plain are among the most extensive wetlands in Northeast China, which harbor rich herbaceous plants such as *Phragmites and Carex* [12]. However, in some parts of the Momoge and Sanjiang wetlands, the degradation of vegetation has been greatly intensified due to grazing and agricultural activity [13,14]. In recent years, the presence of *Carex* tussock and *Phragmites* in the marsh have declined dramatically and disappeared in some parts of Northeast China due to land drainage and the intensification of agriculture [14,15]. To cope with this problem, wetland management departments in China have recently attempted to implement wetland restoration practices by restoring the artificial vegetation and effecting reconstruction by introducing the natural vegetation for recolonization after the recovery of hydrological conditions [14,16,17]. The restoration of wetlands is a promising strategy for reestablishing the ecological structure, function, and biodiversity of the aboveground part of degraded wetlands [17,18]. However, few studies have considered whether the ecological characteristics of the associated below-ground microbial communities respond to deteriorated or restored wetlands [19]. Importantly, little is known about the microbial composition in wetland soil relative to other ecosystems, such as hydrospheres and farmland ecosystems, despite the far-reaching influence of wetlands in controlling biogeochemical cycling at landscape scales [19].

Studies have shown that soil microorganisms play a vital role in plant community dynamics, nutrient availability, soil structure maintenance, and soil biogeochemical cycling [20,21]. In turn, a change in soil conditions and microbial communities leads to a change in the composition of the plant community [20]. Therefore, plant–microbe–soil interactions are very important for the establishment of wetland ecosystems. Previously, studies have reported the effects of ongoing vegetation degradation of wetlands [22] and grasslands [23] on soil respiration. Degraded grasslands show a decline in the carbon content of the soil microbial biomass and microbial functional diversity [24]. Moreover, changes in the composition of soil microorganisms affect carbon loss [25] and enzyme activity variation in the soil [21]. Hartman et al., [19] discovered that bacterial composition and diversity corresponded strongly with soil pH and land use. Further, ecosystem restoration has been shown to alter microbial communities in wetland systems [19,26,27]. The results of a study by Leff et al., [28] indicated that the input of nitrogen and phosphorus to grasslands leads to predictable shifts in the taxonomic and functional traits of soil microbial communities; therefore, these communities are sensitive to nutrient inputs [28]. Nitrogen addition can alter the composition of the bacterial or fungal communities in the soil [29,30,31]. Therefore, understanding the variation in the composition and function of soil microbial communities in wetlands, in response to soil disturbance and human activities such as intensified nitrogen input on agricultural land or grazing wetlands, is favored for ecological restoration.

For this study, our study analyzed soil and plant samples from the Phragmites and Carex marshes that are two typical wetlands of Northeast China located in the Songnen and Sanjiang plains. The natural, degraded, and restored wetlands in these locations were selected for investigation. It was hypothesized that wetlands that were degraded by land drainage and grazing would induce shifts in the relatively abundant bacterial and fungal communities, altering the microbial community composition by increasing the abundance of taxa presumed to be capable of driving nitrogen (N) cycling. Furthermore, the state of the microbial community in restored wetlands may return to that of natural wetlands due to the recovery of soil conditions and flora. Therefore, we examined wetland formation at 16 sites and their distinct effects on the structure and function of the bacterial and fungal community of the soil. The aim of the study was analyzing soil properties, aboveground vegetation, land use, and restoration to obtain a comprehensive survey exploring the major factors affecting soil microbial characteristics. The analysis of these factors would provide a mechanistic understanding of the changes in the microbial community that could be used as an important index to characterize wetland habitats.

## 2. Materials and Methods

### 2.1. Study Sites

**Figure 1:**
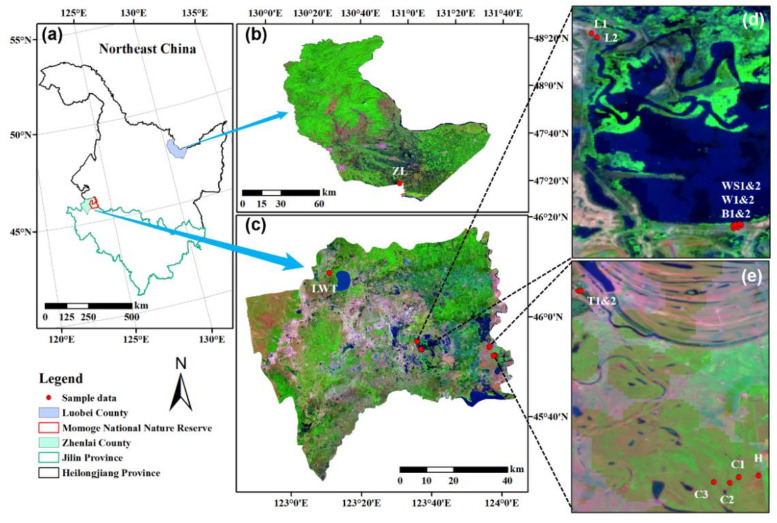
Location of the Momoge wetland in Zhenlai county and the Duluhe wetland in Luobei county (**a**). Remote sensing images from June 2017 of each sampling site are enlarged in (**b**–**e**).

This study was mainly conducted at the Momoge Wetland, one of the two largest wetlands in the Songnen Plain of Jilin Province, China. The climate of the region is a continental monsoon climate, with a mean annual temperature of 4.4 °C. The area has meadow bog soil, dominated by the *Carex*, *Deyeuxia purpurea*, *Scirpus planiculmis*, and *Phragmites australis* plant communities [15]. This area experiences continuous drought and serious salinization/secondary-salinization due to the excessive consumption of water for farmland irrigation in the upper reaches of the Nenjiang River [32]. In recent years, the Chinese government has conducted a wetland restoration project in Momoge, including recovery of the hydrological conditions, vegetation restoration, and construction [32,33,34]. Therefore, this study was conducted at 15 sites in the Momoge Wetland, from two typical marsh sites having the *Phragmites* and *Carex* vegetation types (Figure 1). The sites we selected had a range of land uses encompassing human-disturbed wetlands, restored wetlands, and natural wetlands (Table S1). The additional site studied was located at the Duluhe Wetland, a freshwater wetland in the Sanjiang Plain of Heilongjiang Province, China (Figure 1). This site has *Carex* as the dominant plant and was used as a long-distance reference wetland to compare the similarities and differences relative to the Momoge Carex wetland (Table S1).

### 2.2. Soil and Plant Sampling

Soil samples were collected in the period June–July 2017. In the Phragmites marsh, rhizosphere soil was scraped from plant roots at a depth of 0–15 cm at three sampling locations, with the first plot within each site being randomly selected and the plots selected subsequently were established 5–20 m apart, depending on the area of the site. The size of each plot was 2 m × 2 m. Five soil samples were randomly collected from each plot and mixed as a composite sample. Additionally, in the Carex marsh, three 2 m × 2 m sampling plots were established, and rhizosphere soil was taken from plant roots at a depth of 0–15 cm. After transportation to the laboratory, all soil samples were sieved through a 2 mm mesh to remove visible roots and pebbles and stored at −80 °C and 4 °C for DNA extraction and soil chemical analyses. Before the collection of soil samples, the aboveground plant community was investigated at each study site. Three quadrats within each site were established, and plant coverage, height, and density for specific species were measured in each 1 m × 1 m quadrat. Further, the density of the *Carex* tussock was used for plant analyses in this study. One-way analysis of variance (ANOVA) performed with SPSS^®^ software (v.22.0.0.0) (IBM Corp., Chicago, IL, USA) was used to determine statistical differences of aboveground vegetation properties in the Phragmites and the Carex marsh sampling sites.

### 2.3. Measurement of Soil Physicochemical Properties

Soil pH was measured using a pH meter (Mettler Toledo Corporation, Greifensee, Switzerland) in a water-to-soil mixture ratio of 2.5:1. The total carbon (TC) and total nitrogen (TN) contents were measured with an elemental analyzer (Elementar, Hanau, Germany). The total phosphorus (TP) content was determined using the Sommers-Nelson method [35]. The water content (WC) of the soil samples was identified by dividing the mass difference between fresh and dry soil of each sample by the mass of the dry soil, which was dried at 105 °C for 24 h. Soil salinity, represented by the total soil salt content (TS), was measured using the electric conductivity and dry evaporation methods [36]. Three soil replicates for each sampling site were used for physicochemical analysis, and the average and standard deviation of three replicates determinations were calculated. One-way ANOVA performed with SPSS^®^ software (v.22.0.0.0) (IBM Corp., Chicago, IL, USA) was used to determine statistical differences of soil physicochemical properties in the Phragmites and the Carex marsh sampling sites.

### 2.4. DNA Extraction, High-Throughput Sequencing, and Microbial Community Analysis

DNA was extracted from ~0.5 g of soil (wet weight), using the FastDNA Spin Kit for Soil (MP Biomedicals, Solon, OH, USA) as per the manufacturer’s instructions. Three soil replicates for each sampling site were used for DNA extraction. The quality and concentration of the DNA was assessed by the 260/280 nm and 260/230 nm ratios, which was analyzed using the NanoDrop spectrophotometer (NanoDrop Technologies Inc., Wilmington, DE, USA). The extracted DNA was stored at −20 °C for subsequent amplification and sequencing experiments.

The V4 region of the bacterial 16S rRNA gene was selected for amplification using a pairwise common primer 515F (5′-GTGCCAGCMGCCGCGGTAA-3′) and 806R (5′-GGACTACHVGGGTWTCTAAT-3′) [37]. Additionally, the highly variable internal transcribed spacer 1 (ITS1) region was selected for amplification from soil fungi, using the primer pair ITS5-1737f (5′-GGAAGTAAAAGTCGTAACAAGG-3′) and ITS2-2043r (5′-GCTGCGTTCTTCATCGATGC-3′) [37]. The details for PCR amplification, amplicon purification, library preparation, and processing for Illumina HiSeq sequencing have been described previously [38]. The paired sequences were joined using the Fast Length Adjustment of SHort reads (FLASH) tool [39]. Next, FASTQ files were demultiplexed and quality-filtered using QIIME as per standard protocols [40]. The UPARSE [41] method was used to remove chimeras and classify the sequences into operational taxonomic units (OTUs) based on a 97% similarity. Taxonomic assignments of bacteria at the order level and fungi at the genus level were performed using the SILVA database [42] as reference. Alpha and beta diversity was calculated using the QIIME and R software (v.4.0.2) [43] based on the normalized data. Shifts in the microbial community composition were visualized via non-metric multidimensional scaling (NMDS) ordinations based on the Bray-Curtis dissimilarity matrix. The *t*-test was used to compare the means of relative abundance of bacteria and fungi between the soil groups. Pairwise comparisons were further verified by ANOSIM and PERMANOVA tests for the structure of soil communities between samples from the Phragmites or Carex sites, respectively. The *t*-test was performed with SPSS^®^ software (v.22.0.0.0) (IBM Corp., Chicago, IL, USA), and the rest of the statistical analyses were performed on the Galaxy pipeline (http://159.226.240.74:8080, 10-06-2020). Differences were considered statistically significant at a value of *p* < 0.05 when distinguishing the microbial structure in the compared groups. All of the sequence data have been submitted to the GenBank Sequence Read Archives (http://www.ncbi.nlm.nih.gov) under BioProject ID PRJNA660301 and PRJNA660325.

### 2.5. GeoChip Analysis

A new generation of functional gene arrays (GeoChip 5.0) was used to analyze the functional diversity, structure, metabolic activity or potential, and dynamic changes in microbial communities [44]. The GeoChip 5.0 array contains key target genes involved in carbon, nitrogen, phosphate, and sulfur geochemical cycles in addition to other functional processes [44]. We selected three typical groups of soil samples each from the Phragmites and the Carex marshes for GeoChip analysis (Table S1). Approximately 500 ng of the purified soil DNA was labeled with Cy-3 and hybridized onto the GeoChip array, as described previously [44]. After hybridization, the GeoChip slides were washed with buffers to remove unbound DNA. The arrays were scanned with a NimbleGen MS200 scanner (Roche, Madison, WI, USA), and the image data were extracted using the Agilent Feature Extraction program [45]. Raw data were analyzed using a data analysis pipeline, as described previously [45,46]. Briefly, poor quality spots with a signal-to-noise ratio (SNR = signal mean-background mean/background standard deviation) of less than 2.0 were removed before statistical analysis. Next, the hybridization signals were normalized by a relative abundance method and a natural logarithmic transformation [46,47].

The significance of differences in the abundance of microbial phylogenetic and functional genes in a comparison of samples from natural vs. degraded, restored vs. degraded, and severely vs. lightly degraded wetlands were calculated using the unpaired *t*-test and the response ratio (RR). The RR is a common effect size metric used to quantify the outcome of experiments for ecological meta-analysis [48,49], by calculating the log proportional change between the means of a treatment and control group (e.g., natural vs. degraded wetlands in this study) as follows (1):

(1) $$RR=\ln\left(\bar{X_{t}}/\bar{X_{c}}\right)$$

where, $\bar{X_{t}}$ and $\bar{X_{c}}$ are the means of the concerned variables in the treatment and control groups, respectively. The variance (ν) is approximately equal to the following Equation (2):

(2) $$\nu =\frac{s_{t}^{2}}{n_{t}{\bar{X}}_{t}^{2}}+\frac{s_{c}^{2}}{n_{c}{\bar{X}}_{c}^{2}}$$

where $n_{t}$ and $n_{c}$ are the sample sizes for the treatment and control groups, respectively, and $s_{t}$ and $s_{c}$ are the standard deviations for all comparisons in the treatment and control groups, respectively. In the meta-analysis, the weighted RR ($RR_{++}$) is calculated from individual $RR_{ij}$ ($i=1,\;2,\;\ldots ,\;m;j=1,\;2,\;\ldots ,\;k_{i}$) for more accurate estimates (Equation (3)) [50], where m is the number of groups, $k_{i}$ is the number of comparisons in the ith group, and $w_{ij}$ is the weighting function calculated based on the reciprocal of the variance (Equation (4)).

(3) $$RR_{++}=\frac{\sum_{i=1}^{m}\sum_{j=1}^{k_{i}}w_{ij}RR_{ij}}{\sum_{i=1}^{m}\sum_{j=1}^{k_{i}}w_{ij}}$$

(4) $$w_{ij}=\frac{1}{v}$$

The standard error of $RR_{++}$ is calculated as follows (5):

(5) $$S\left(RR_{++}\right)=\sqrt{\frac{1}{\sum_{i=1}^{m}\sum_{j=1}^{k_{i}}w_{ij}}}$$

The differences in the functional microbial community composition among soil groups were analyzed by NMDS based on the Bray-Curtis dissimilarity matrix. A canonical correspondence analysis (CCA) and variation partitioning analysis (VPA) were performed to explore the correlations between the functional gene structure of microbial communities, soil, and vegetation variables. Before CCA and VPA modeling, all soil or vegetation variables were included in the Mantel test, and the redundant variables were removed by using manual forward selection with the Monte Carlo permutation test with 999 permutations (*p* ≥ 0.05) and a variance inflation factor (VIF) ≥ 20. All statistical analyses were conducted using the Galaxy pipeline (http://159.226.240.74:8080, 10-06-2020) [51,52].

## 3. Results

### 3.1. Changes in the Vegetational and Edaphic Variables of Different Types of Wetlands

**Figure 2:**
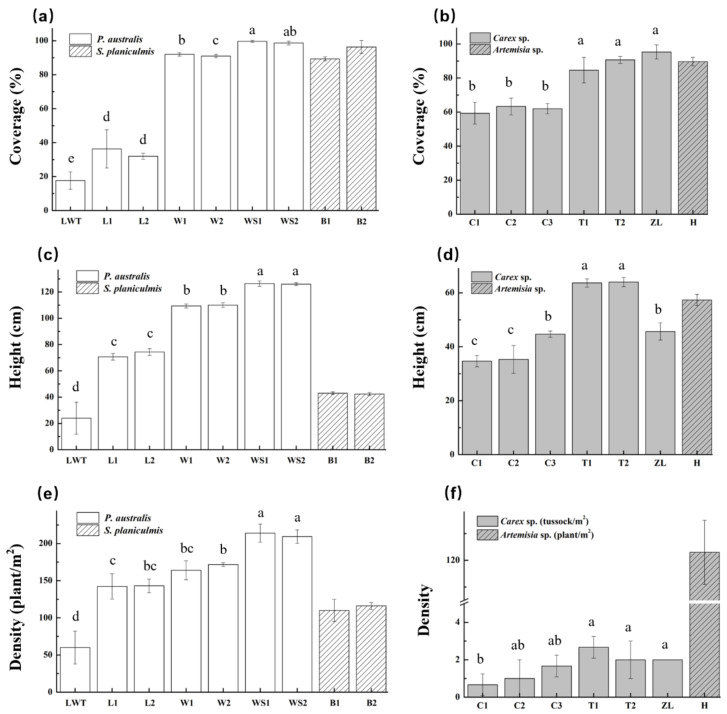
(**a**) Coverage of aboveground vegetation in the Phragmites marsh sampling sites; (**b**) coverage of aboveground vegetation in the Carex marsh sampling sites; (**c**) height of aboveground vegetation in the Phragmites marsh sampling sites; (**d**) height of aboveground vegetation in the Carex marsh sampling sites; (**e**) density of aboveground vegetation in the Phragmites marsh sampling sites; (**f**) density of aboveground vegetation in the Carex marsh sampling sites. Error bars represent one standard deviation of the mean (*n* = 3). Different letters above the bars indicate significant differences (*p* < 0.05) among different sites, calculated by one-way ANOVA.

Changes in the aboveground vegetation in the Phragmites and Carex marshes were similar (Figure 2), as plant coverage and height decreased sharply in the degraded wetlands. In the Phragmites marsh, the degraded sites L1, L2, and LWT showed a step-down trend for aboveground *Phragmites* coverage, height, and density (Figure 2a,c,e). Therefore, the sampling sites LWT and L (L1 and L2) were classified according to plant factors as severely and lightly degraded Phragmites wetlands, respectively. In the restored wetland (via *P. australis* vegetation restoration in the Momoge National Nature Reserve), the aboveground plant factors were higher as the *Phragmites* sp. were closer to the lake (*p* < 0.05). Similarly, in the Carex marsh, *Carex* sp. in natural wetlands (T1, T2, and ZL) grew better than in the degraded marsh (C1, C2, and C3) (Figure 2b,d). However, in the case of mesophyte invasion in *Carex* sp. marsh sites, plant factors such as the *Carex* sp. coverage and *Carex* tussock density were not significantly different in the degraded Carex marsh sites (C1, C2, and C3; *p* > 0.05; Figure 2b,d). However, the plant height of *Carex* sp. was significantly reduced near the boundaries of the invasive *Artemisia* sp. and the Carex marsh (*p* < 0.05, Figure 2f).

The soil characteristics of the Phragmites and Carex marshes are displayed in Table 1 and Table 2. All the samples of Phragmites marsh soil were alkaline or slightly alkaline, and the severely degraded site LWT exhibited a significantly strong alkaline nature (pH 9.48 ± 0.29). Further, the site LWT presented a significantly high TN content (1.51 ± 0.45 g/kg), while TN content at other Phragmites marsh sites ranged from 0.41–1.14 g/kg. The TC content was significantly increased in all the degraded Phragmites wetlands sampled (LWT, L1, and L2; *p* < 0.05). The TP content was lowest (0.20–0.34 g/kg) in the degraded Phragmites wetlands (LWT, L1, and L2), and was highest in the restored wetlands (0.47–0.93 g/kg). Moreover, the restored Phragmites marsh showed increased WC in the soil samples (W1, W2, WS1, and WS2) (Table 1).

The degraded and natural Carex marsh soils were neutral or slightly acidic (Table 2). In the Carex marsh group, the sampling site C1, the boundaries of the *Artemisia* sp., and the Carex marsh showed the highest TN (4.69 ± 1.08 g/kg) and TC (47.24 ± 5.61 g/kg) content (*p* < 0.05). The TP and TS contents did not differ significantly between degraded and natural Carex wetlands. Notably, the soil WC was significantly increased in the natural Carex wetlands (T1 and ZL) (*p* < 0.05).

### 3.2. Comparison of the Taxonomic Composition of Bacterial and Fungal Communities among Wetlands

DNA sequencing using the Illumina HiSeq platform yielded 4,141,833 high-quality bacterial 16S rRNA gene sequences and 4,339,212 fungal ITS gene sequences from all samples from the 16 wetlands sites (3 replicates for each site). After random resampling, each sample generated 16,703 bacterial and 14,355 fungal OTUs, identified with a 97% similarity cutoff. An α-diversity analysis indicated that bacterial diversity in the Phragmites (Table 3) and Carex marsh soils (Table 4) was significantly decreased with increasing wetland degradation. However, the Simpson index of the Carex marsh soil was an exception (Table 3). The large spread of the W2 was likely due to the obvious difference in soil WC (30.90% ± 8.20%) of each sample in this site. The heterogeneity of soil sample existed in sampling plots because Phragmites wetlands are at the interface of aquatic and terrestrial ecosystems. The α-diversity of the fungal community and the Shannon and Simpson indices presented a similar trend for the bacterial community (Table 3 and Table 4).

**Figure 3:**
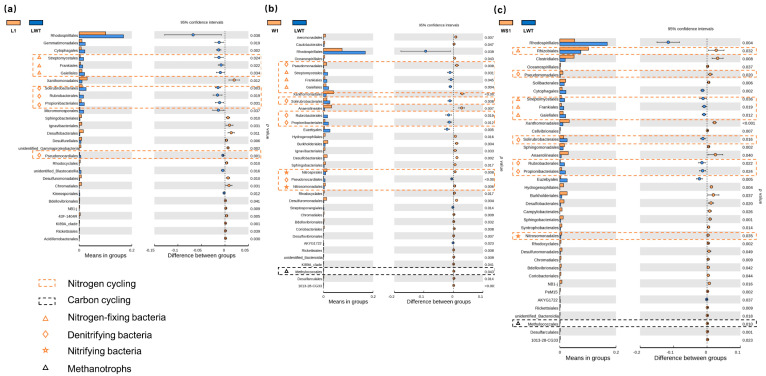
A comparison of the relative abundance of two soil bacterial groups between restored and degraded Phragmites wetlands at the level of bacterial orders. (**a**) comparison between L1 and LWT sites; (**b**) comparison between W1 and LWT sites; (**c**) comparison between WS1 and LWT sites. The significance of the differences between the two groups was determined using the *t*-test with 95% confidence intervals. The orange and black dashed boxes represent typical bacteria involved in nitrogen and carbon cycling, respectively. Nitrogen-fixing, denitrifying, and nitrifying bacteria are marked by orange triangles, diamonds, and pentacles, respectively. Methanotrophs are marked by black triangles.

**Figure 4:**
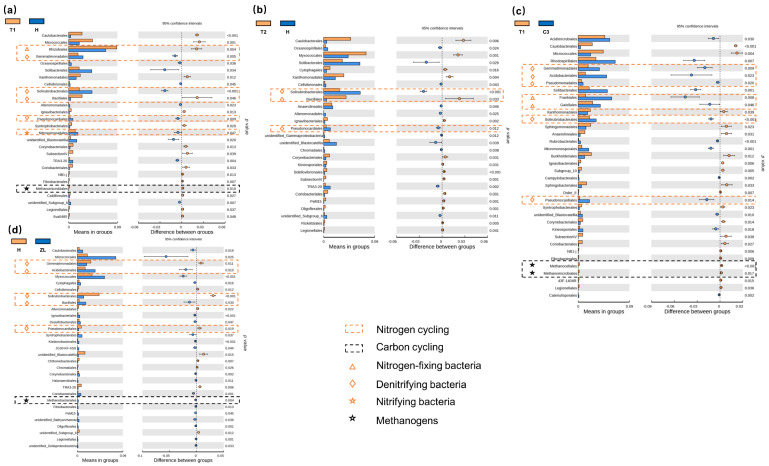
A comparison of the relative abundance of two soil bacterial groups between the natural and degraded Carex wetlands at the level of bacterial orders. (**a**) comparison between T1 and H sites; (**b**) comparison between T2 and H sites; (**c**) comparison between T1 and C3 sites; (**d**) comparison between H and ZL sites. The significance of differences between two groups was determined using the *t*-test with 95% confidence intervals. The orange and black dashed boxes represent typical bacteria involved in nitrogen and carbon cycling, respectively. Nitrogen-fixing, denitrifying, and nitrifying bacteria are marked by orange triangles, diamonds, and pentacles, respectively. Methanogens are marked by black triangles.

In the bacterial communities, the generally dominant phyla present in all sites were the *Proteobacteria* and *Actinobacteria* (Figure S1a). However, the abundance of *Proteobacteria* and *Actinobacteria* appeared to decline and increase, respectively, in all degraded Phragmites and Carex wetlands tested (LWT, L1, L2, H, C1-C3), compared with other natural and restored wetlands (Figure S1a). Simultaneously, we compared pairs of samples between the degraded and restored/natural wetlands at the level of bacterial orders and examined the bacterial diversity between the two soil groups (Figure 3 and Figure 4). Notably, the relative abundance of methanotrophs was significantly increased (*p* < 0.05) in the restored Phragmites wetlands (Figure 3b,c). In addition, bacteria participating in soil nitrogen cycling were most abundant in the LWT, with some exceptions (Figure 3). In the Carex marsh sites, the relative abundance of methanogens (including *Methanomicrobiales*, *Methanocellales*, and *Methanobacteriales*) was significantly increased (*p* < 0.05) in natural wetlands (Figure 4a,c,d). The abundance of bacteria involved in the fixing of atmospheric nitrogen, nitrification, and denitrification showed no unified change between degraded and natural Carex sites.

**Figure 5:**
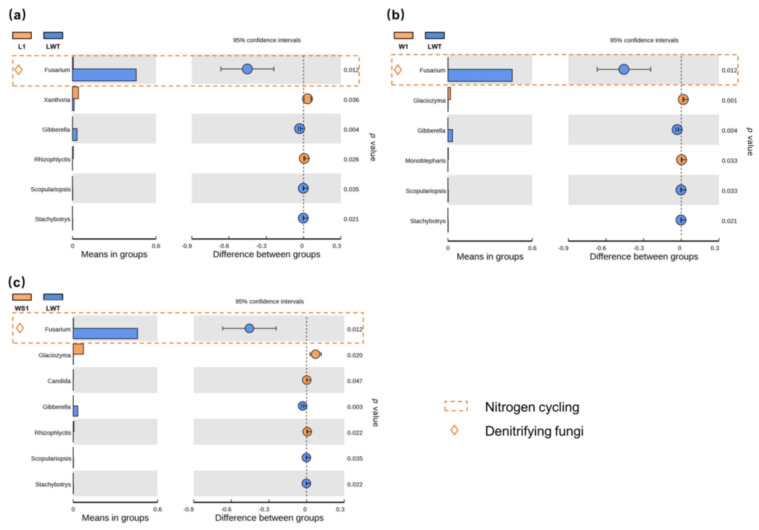
A comparison of the relative abundance of two soil fungal groups between restored and degraded Phragmites wetlands at the level of fungal genera. (**a**) comparison between L1 and LWT sites; (**b**) comparison between W1 and LWT sites; (**c**) comparison between WS1 and LWT sites. The significance of differences between the two groups was determined by a *t*-test with 95% confidence intervals. The orange dashed boxes represent typical bacteria concerned in nitrogen cycling. Denitrifying fungi are marked by orange diamonds.

**Figure 6:**
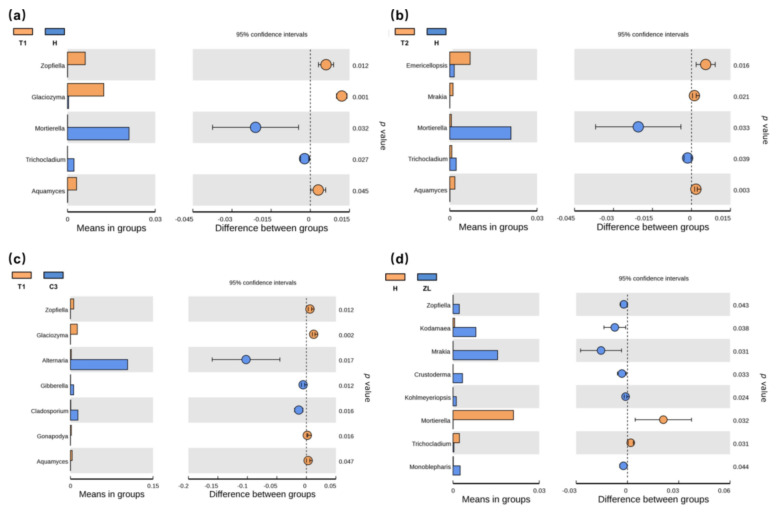
A comparison of the relative abundance of two soil fungal groups between natural and degraded Carex wetlands at the level of fungal genera. (**a**) comparison between T1 and H sites; (**b**) comparison between T2 and H sites; (**c**) comparison between T1 and C3 sites; (**d**) comparison between H and ZL sites. The significance of differences between the two groups was determined by a *t*-test with 95% confidence intervals.

As with bacterial communities, the abundance of fungal phyla was changed among different wetlands (Figure S1b). The OTUs assigned to the phylogenetic taxa showed *Ascomycota* as the predominant fungal phyla. We compared pairs of soil samples between degraded and restored/natural wetlands at the fungal genus level (Figure 5 and Figure 6). The proportion of *Fusarium*, recognized as phytopathogens and denitrifiers [53] was increased substantially at the LWT site (*p* < 0.05), relative to other Phragmites wetlands. In the Carex marsh sites, *Zopfiella*, *Glaciozyma*, and *Aquamyces* were significantly enriched relative to the invasive *Artemisia* sp. (*p* < 0.05), and the occurrence of fungi from the genera *Mortierella* and *Trichocladium* was significantly increased (*p* < 0.05) relative to the *Artemisia* sp. (Figure 6).

**Figure 7:**
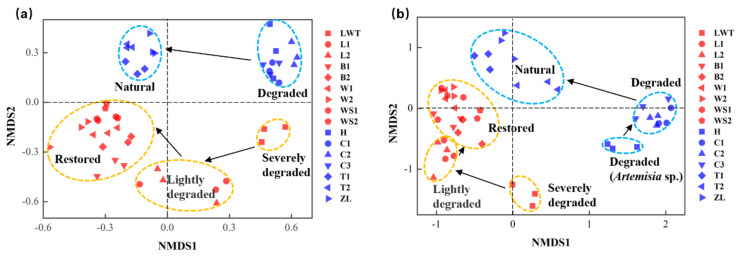
NMDS analysis of the soil bacterial (**a**) and fungal (**b**) community in the Phragmites and the Carex marsh sampling sites. The red and blue symbols represent the Phragmites and Carex sites in the NMDS analysis, respectively, and the black arrows indicate the bacterial or fungal composition shift in the different wetland sites.

The NMDS analysis, based on 16S rRNA and ITS gene sequencing, showed distinct bacterial and fungal communities in the sampled sites, with microbial clusters around the Phragmites or Carex marshes, and in the natural, degraded, and restored wetland regions (Figure 7). Samples in degraded wetlands showed a distinct bacterial community composition relative to the natural and restored wetlands. The B and H sites, with different aboveground vegetation in the Phragmites and Carex marshes, respectively (Table S1 and Figure 2), occurred in clusters in the W and WS restored Phragmites sites and the degraded Carex site, respectively (Figure 7a). In terms of fungal community structure, the H soil samples, with Artemisia sp. occurring aboveground, were distinct in the degraded Carex sites (C) (Figure 7b). Interestingly, the samples in the first scaled dimension showed a more similar bacterial community composition between restored Phragmites sites and the natural Carex sites, and between severely degraded Phragmites sites and degraded Carex sites. Unlike the bacterial communities, samples in the first scaled dimension showed a more similar fungal community composition between restored and lightly degraded Phragmites sites than in any other pair. Further, the most significant bacterial and fungal community composition change was observed in severely degraded sites (LWT) (Tables S2 and S3, *p* < 0.05) at Phragmites sites, though the community gradually returned to a better state with improving wetland conditions (Figure 7). These results were further verified by ANOSIM and PERMANOVA tests (Tables S2 and S3).

### 3.3. Comparison of Microbial C/N Cycling Genes among Wetlands

**Figure 8:**
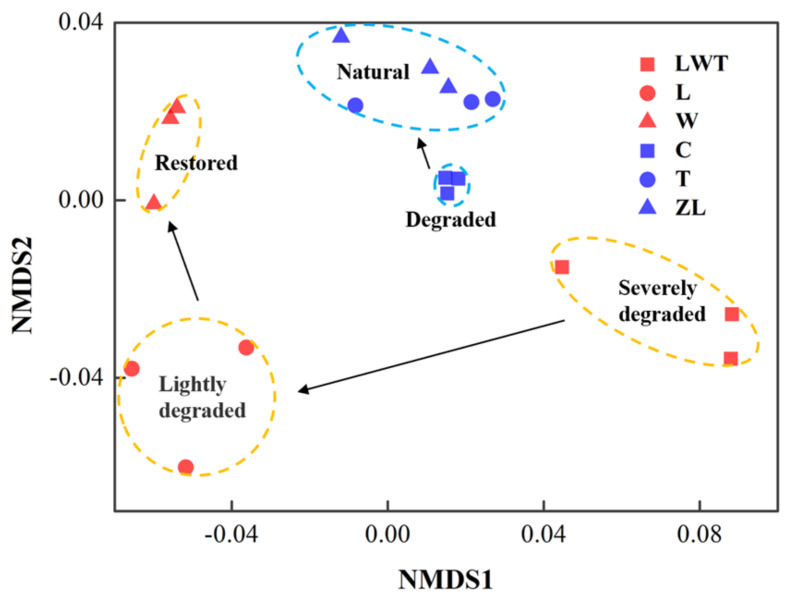
NMDS analysis based on the results of the GeoChip microarray showing functional microbial gene abundance. The red and blue symbols represent selected samples from the Phragmites and Carex sites, respectively, and the black arrows indicate a functional shift in microbial composition in different wetland sites.

DNA extracted from the selected representative soil samples was analyzed using the GeoChip 5.0 array. A total of 865,131 detected genes distributed across 1039 functional gene families were detected. NMDS analysis based on functional gene composition as indicated in Figure 8 illustrates that the samples showed grouping according to the Phragmites or Carex sites, and samples from degraded wetlands were distinct from those from the natural or restored wetlands. Additionally, a comparison of functional community structure dissimilarity using the ANOSIM test based on the Bray-Curtis index revealed a significant difference among the LWT, L, and W sites (*p* = 0.004), and among C, T, and ZL sites (*p* = 0.005, Figure S2). Interestingly, samples from the restored Phragmites sites (W) and natural Carex sites (T and ZL) were more similar along the second scaled dimension than other sample pairs, and the functional community gradually returned to a better state with improving Phragmites wetland conditions as well (Figure 8).

**Figure 9:**
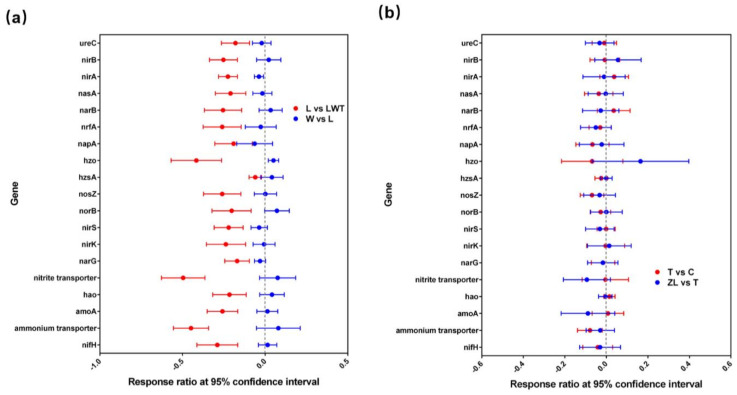
The response of N cycling-related genes to degraded and restored/natural wetland sites as per GeoChip data. (**a**) RRs are presented as the difference between lightly degraded and severely degraded Phragmites marsh sites (red symbols), and between restored and lightly degraded Phragmites marsh sites (blue symbols). (**b**) RRs are presented as the difference between the natural and degraded Carex marsh sites (red symbols), and between the two natural Carex marsh sites (blue symbols). The RR is considered significant when the 95% confidence interval (presented as error bars) does not overlap with 0. The abbreviations present restored (W), lightly degraded (L), and severely degraded (LWT) *Phragmites* marsh sites, and the natural (T, ZL) and degraded (C) *Carex* marsh sites, respectively.

As the degraded wetland sites showed a significantly high TN content, we investigated key functional genes associated with N cycling. GeoChip data showed that a number of N cycling genes showed increased abundance in response to the degradation of Phragmites wetlands (Figure 9 and Figure S3). The abundance of 95% of the N cycling genes was significantly increased in microbes from the severely degraded Phragmites sites (LWT). These genes showed enrichment for the N_2_ fixation (*nifH*), ammonium transporter, nitrification (*amoA*, *hao*), nitrite transporter, denitrification (*narG*, *nirK*, *nirS*, *norB*, *nosZ*), anaerobic ammonium oxidation (anammox; *hzo*), dissimilatory N reduction to ammonium (*napA*, *nrfA*), assimilatory N reduction (*nasA*, *narB*, *nirA*, *nirB*), and the ammonification genes (*ureC*) (*p* < 0.05, Figure S3a), except for the *hzsA* gene which is involved in anammox. The grazed LWT sites showed a significantly higher abundance of N cycling genes relative to the lightly degraded Phragmites wetlands (L), and the restored Phragmites sites (W) showed significantly decreased abundance of *nirA* and the increased abundance of anammox genes *hzo* (*p* < 0.05); however, compared with L sites, the abundance of other N cycling genes was not affected (Figure 9a). Gene abundance changes in the *hzo* genes appeared to be related to the restored hydrologic conditions in W sites. In contrast, the abundances of two genes involved in ammonium transporter and denitrification (*nosZ*) were significantly altered in samples from degraded Carex sites (C), compared with samples from natural wetlands (T and ZL) (Figure 9b and Figure S3b).

**Figure 10:**
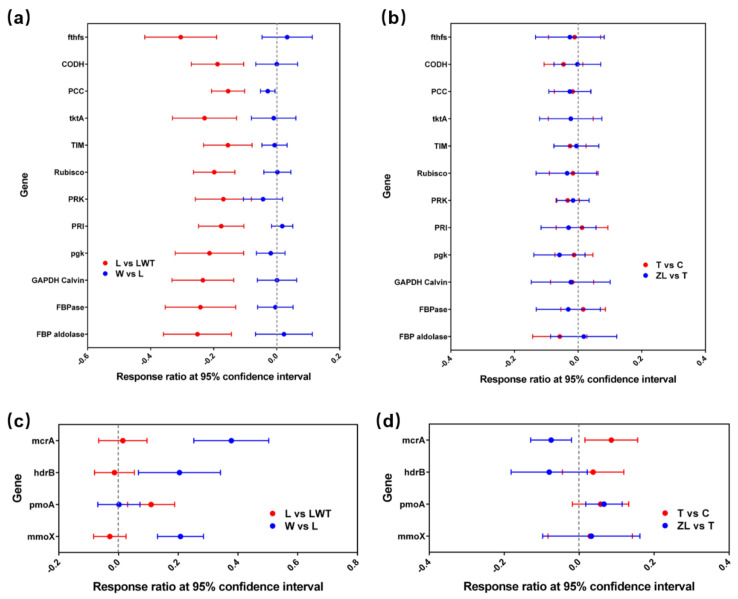
Response of C fixation and methane metabolism-related genes to degraded and restored/natural wetland sites according to GeoChip data. RRs of C fixation (**a**) and methane metabolism (**c**) genes are presented as the difference between lightly degraded and severely degraded Phragmites marsh sites (red symbols), and between restored and lightly degraded Phragmites marsh sites (blue symbols). RRs of C fixation (**b**) and methane metabolism (**d**) genes are presented as the difference between natural and degraded Carex marsh sites (red symbols), and between two natural Carex marsh sites (blue symbols). The RR is considered significant when the 95% confidence interval (presented as error bars) does not overlap with 0. The abbreviations present restored (W), lightly degraded (L), and severely degraded (LWT) *Phragmites* marsh sites, and the natural (T, ZL) and degraded (C) *Carex* marsh sites, respectively.

Next, we analyzed the abundance of key genes associated with C fixation and methane metabolism (Figure 10 and Figure S4), and the results showed that the abundance of most genes related to C fixation was significantly increased in samples from LWT sites (*p* < 0.05) (Figure S4a). Moreover, the abundance of the *PCC* gene encoding propionyl-CoA carboxylase was significantly decreased in samples from the restored Phragmites wetlands, relative to those from the degraded sites (*p* < 0.05). In the Carex wetlands, the abundance of C fixation genes was not significantly altered in samples from natural and degraded sites (Figure 10b).

We detected four key functional genes related to methane metabolism, including *mcrA* and *hdrB* as indicators of methanogenesis, and *pmoA* and *mmoX* that encode methane monooxygenases for methane consumption. Notably, an RR analysis indicated that these gene categories related to methane metabolism were sensitive to the condition of the Phragmites wetlands. The abundance of *mcrA*, *hdrB*, and *mmoX* genes was significantly increased in samples from restored Phragmites wetlands (W), compared to samples from lightly degraded sites (L) (*p* < 0.05; Figure 10c). Moreover, the abundance of *pmoA* in samples from LWT sites was lower than in samples from other Phragmites sites (*p* < 0.05), which was consistent with the results of the influence of degradation on the methanotroph community composition as per data from 16S rRNA sequencing (Figure 3 and Figure 10c). The abundance of the *mcrA* genes, which was mainly derived from methanogenic archaea, appeared to be sensitive to degradation, as the abundance of these genes was significantly reduced in samples from degraded sites from the Carex wetlands (Figure 10d).

**Figure 11:**
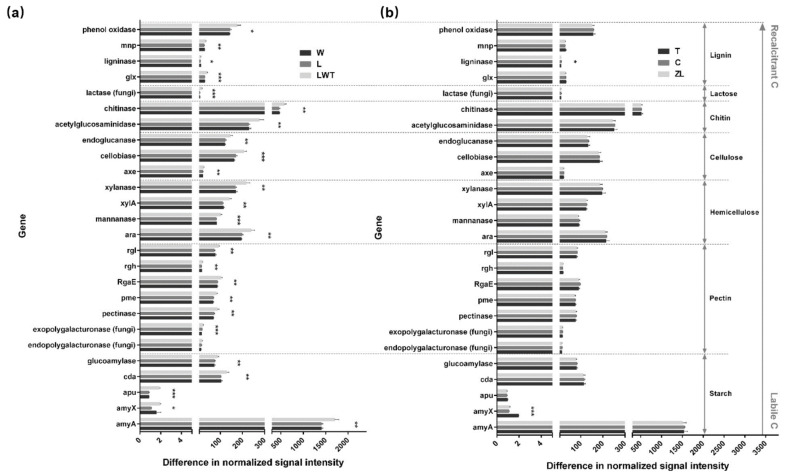
Normalized GeoChip signal intensities of genes involved in C decomposition in the restored (W), lightly degraded (L), and severely degraded (LWT) Phragmites marsh sites (**a**), and the natural (T, ZL) and degraded (C) Carex marsh sites (**b**), respectively. Error bars represent one standard deviation of the mean (*n* = 3). ***, *p* < 0.001, **, *p* < 0.01, *, *p* < 0.05, based on the unpaired *t*-test. The complexity of carbon is ordered from labile to recalcitrant.

Further, the abundance of 96% of the genes associated with C degradation was significantly increased in response to severe degradation at Phragmites sites, including genes related to the degradation of starch, pectin, hemicellulose, cellulose, chitin, lactose, and lignin (Figure 11). The samples from restored Phragmites sites (W) showed a significant upregulation of the *amyX* gene that encodes an alpha-amylase, the *rgh* gene involved in pectin degradation, and the gene coding for ligninase (*p* < 0.05). The abundance of genes from three gene families encoding exopolygalacturonase (fungi), pectinase, and cellobiase, which were associated with C decomposition in W soils was lower than at L sites (*p* < 0.05; Figure S5a). The abundance of 12% of the C decomposition genes was significantly increased at T sites in samples from the Carex marshes, than in samples from the degraded Carex sites. Furthermore, the abundance of these genes (*amyX*, *rgh*, and *mnp* - lignin degradation) was similar in samples from the restored Phragmites sites (W) (Figure S5b).

### 3.4. Association between the Functional Structure of the Microbial Community and Environmental Variables

We used multiple statistical methods to explore the association between several environmental variables (soil physicochemical parameters and aboveground vegetation parameters) and the functional structure of microbial communities, as degradation affects both factors. First, we analyzed the correlation between total environmental factors or vegetation properties and microbial communities using Mantel tests (Tables S4 and S5). In Phragmites marshes, the aboveground plant coverage was found to have a significant effect on the composition of the bacterial and fungal communities (all *r* > 0.60, all *p* < 0.001), and the TN and TC were found to be important environmental factors driving variation in microbial communities (*p* < 0.05). In the Carex marshes, results of the Mantel tests indicated that the WC of soil had a significant impact on the microbial community structure (all *r* > 0.45, all *p* < 0.001). Furthermore, the pH and vegetation variables (vegetation species, coverage, and diversity) were found to be major factors influencing the microbial functional community structure (all *r* > 0.30, all *p* < 0.001), when we performed Geochip data in Phragmites and Carex sites together (Table S6). Considering that different wetland types may affect the functional structure of microbial communities via different mechanisms, we analyzed the Phragmites and Carex sites separately using Mantel tests (Table S7). The results suggested that the microbial community structure was significantly correlated with the pH, TN, and vegetation variables (vegetation coverage and diversity) in Phragmites marshes (*p* < 0.05), whereas the WC and Carex coverage were found to have a significant effect on microbial communities in Carex marshes (*p* < 0.05).

Next, we used the Monte Carlo permutation test (*p* < 0.05) and a VIF (<20) analysis for the step-wise removal of redundant variables for a CCA. Correlations between the selected soil and vegetation variables, microbial community composition, and microbial functional genes were estimated by CCA modeling and VPA. The results of the CCA models, including the selected environmental and vegetation variables, were significant for the microbial taxonomic composition (*p* < 0.05), which was also supported by Mantel tests (Figure S6). In the samples from the Phragmites marshes, the VPA results showed that TN, TC, TP, aboveground plant coverage and density, and their interaction explained 33.62% of the variation in bacterial community structure, and 30.57% of the variation in the fungal community structure (Figure S6). In the Carex marshes, the pH, TN, TC, WC, and plant variables were major factors contributing to 52.56% of the total variation in bacterial community structure (Figure S6c,d). In addition, the VPA results showed that the amount of variance explained by edaphic factors (pH and WC, 14.09%) was higher than that explained by vegetation factors (7.02%) in the fungal community structure (Figure S6h).

**Figure 12:**
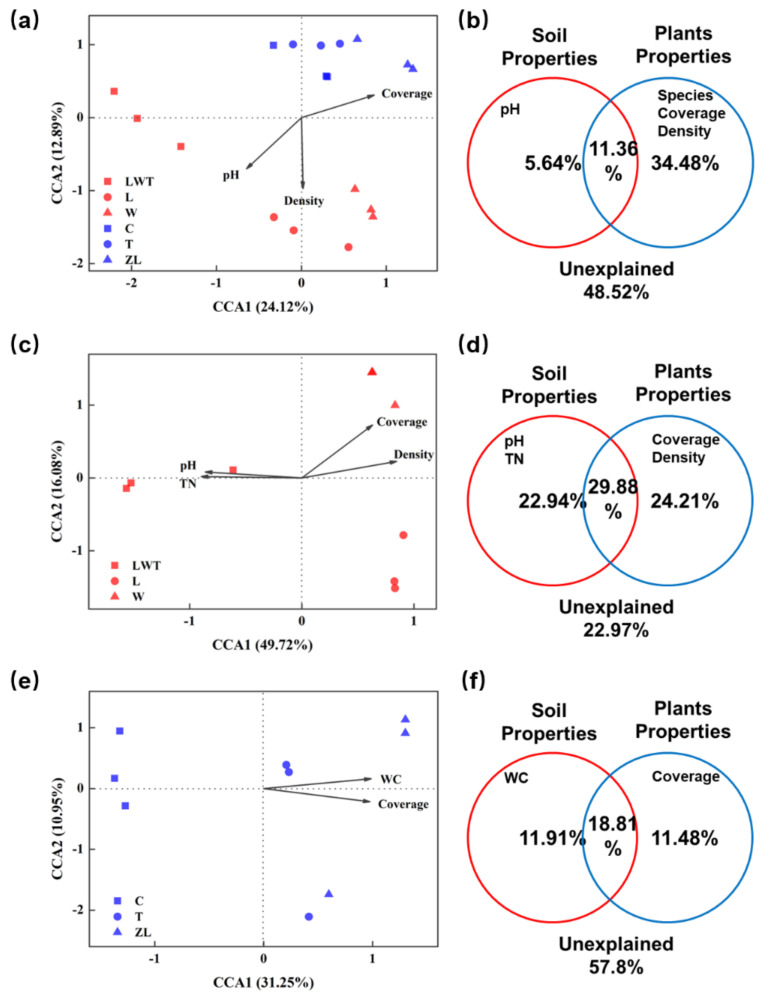
CCA and VPA analysis based on selected environmental variables and GeoChip microarray detected N and C cycling-related functional gene abundances in samples from Phragmites and Carex sites (**a**,**b**), selected samples from Phragmites sites alone (**c**,**d**) and Carex sites alone (**e**,**f**). The percentage values of axis 1 and 2 in CCA indicate the percentage of variation explained by the corresponding axes. Environmental variables in VPA are divided into groups of soil and plant properties. The variance unexplained by the tested variable groups is indicated in the VPA figure.

Further, we compared the individual contributions of environmental factors and vegetation factors in explaining the microbial functional community structure in different wetland types. The VPA clearly showed that soil pH had an exclusive effect on the functional microbial composition involved in C and N cycling from all samples from Phragmites and Carex marshes, after accounting for variable categories explaining a higher proportion of variance (34.48%), which was also corroborated by the CCA and Mantel tests (Figure 12a,b). Considering that the functional microbial community structure might be affected by different mechanisms, we performed a separated constrained ordination analysis for Phragmites and Carex marshes alone. In the analysis for Phragmites sites, a total of 77.03% of the variation in the microbial community could be explained by the selected vegetation and soil variables (Figure 12d). The VPA results also revealed that the vegetation and environmental conditions at Phragmites sites shaped the variation in the functional microbial community composition involved in C and N cycling, more significantly than the variation in community composition (Figure 12 and Figure S7). Further, the results of the CCA and Mantel tests indicated that among the measured variables, vegetation density and TN were the most important drivers of the variation in C/N functional composition (the highest Mantel r value; Figure 12 and Table S7). In Carex marshes, vegetation coverage and soil moisture content together contributed 42.2% of the total variance in functional microbial community composition involved in C and N cycling (Figure 12f).

## 4. Discussion

Wetlands are the third major ecosystem type on Earth and have unique hydrological and soil conditions and biodiversity [54]. However, they are highly sensitive to and threatened by human activities [19,55,56]. The Northeast region has one of the most extensive wetland areas in China, and with the ever-growing human demand for water and food, heavy grazing, and land reclamation has severe adverse environmental impacts in this wetland region, resulting in a decline in the wetland flora, biodiversity, populations, and coverage [12,18]. Although the response of plant communities to changes in wetland conditions has been well-studied, the response of invisible microorganisms that are important drivers for wetland ecosystem processes remains poorly understood. In this study, we performed high-throughput sequencing and GeoChip array hybridizations to analyze microbial characteristics in different wetland types and to dissect the interactions between the functional microbial community structure, ecological responses, and biogeochemical processes.

### 4.1. Relationship between Microbial Diversity and Ecological Vulnerability in Wetlands

Results from previous studies indicate that plant diversity, together with plant species, interactively influences ecosystem functions [57,58]. However, the diversity and composition of soil microorganisms are also related to ecosystem processes and functions [59,60]. This is consistent with the results from our experiments, which show that microbial diversity plays a key role in determining the ecological responses of wetland ecosystems to current environmental change.

The results of the α-diversity analysis for bacterial and fungal communities indicated that microbial diversity showed a rising trend along with the gradually improving Phragmites wetland conditions (Table 3 and Table 4). Therefore, the diversity of soil microbes and plant communities, which are the two key functional groups that form the basis of wetland ecosystems, and the interactions with abiotic soil factors, may have major consequences on the ecological vulnerability of wetlands. Previous studies have shown that the responses of ecosystems (sensitivity and resilience) to disturbances are regulated by extensive interactions between the vegetation structure, water resources, and geographic and geomorphic conditions [61,62,63]. Consistently, there was a significant decrease in soil moisture content, aboveground vegetation (*Carex* sp.) properties, and microbial diversity at C sites, compared with natural Carex sites. These results were indicative of drought stress, resulting from the human demand for water for field irrigation and increasing climate uncertainty, and causing mesophyte invasion. Furthermore, the microbial communities of *Artemisia* invaded sites clustered around the degraded Carex sites, suggesting that microbial composition and diversity from sites responded earlier than aboveground plants. Notably, the weak site effects of T and ZL sites were surprising, given the long geographic distance between the two sampling sites. It is likely that wetland ecosystem functions may be predicted by the structure of microbial communities, although it is generally accepted that plant community composition is key for ecosystem assessment, and recent studies are consistent with this hypothesis [64,65]. The native Carex tussock exists aboveground and maintains growth in the degraded Carex sites, whereas microbial groups in the soil microcosm were sensitive to the disturbance immediately. The decrease in microbial diversity and change in composition was not conducive to the development of Carex wetlands and appeared to contribute to the transition to a more vulnerable wetland ecosystem without the lost microbial functionality of degraded wetlands [19].

### 4.2. Importance of Microorganisms in Wetland Biogeochemical Processes

Accumulating evidence has shown that soil microbial communities, as important engines, are crucial for driving biogeochemical cycles and changing below-ground processes linked to the functioning of the ecosystem [46,66,67,68]. Therefore, simply documenting how microbial communities shift in composition might not reveal the interconnections between biogeochemical processes and environmental perturbations. As wetlands are regions threatened by human activities leading to variations in the ecosystem, an understanding of how the below-ground microbial communities respond to human intervention is a central issue for wetland ecosystems. To address this, we performed GeoChip array hybridizations to dissect the functional diversity and structure, and dynamic changes in microbial communities across three typical sites from Phragmites and Carex marshes, respectively.

The results showed that the abundance of a number of N cycling genes was increased in samples from LWT sites, the severely degraded Phragmites wetlands. The LWT sampling sites were the major land used for livestock grazing (Table S1), and the grazing effect on vegetation was apparent, as indicated by the reduction in aboveground *Phragmites* properties (Figure 2). Grazing on wetlands significantly changed the overall functional gene abundance during nitrogen cycling, including the abundance of genes for N fixation, mineralization, nitrification, denitrification, and assimilatory/dissimilatory N reduction, compared with other Phragmites sites (Figure 9). In addition, the abundance of the soil bacterial communities related to N cycling was increased (Figure S2), which was consistent with changes in the abundance of the functionally associated genes. The proportion of *Fusarium*, recognized as a denitrifier [53], was significantly increased at the LWT site (*p* < 0.05; Figure 5). The effect of grazing on microbial community structure has been addressed in several previous studies [46,69,70,71]; however, most of these studies focused on the influence of grazing on grasslands. Little is known about the effect of grazing on wetland below-ground biodiversity and functional structure, although wetlands are one of the main hot spots for regulating climate change and are highly sensitive to anthropogenic perturbation. Grazing decreased the proportion of the Phragmites area, which was occupied by *Leymus chinensis* in the grazed LWT territories. However, Le Roux et al., [72] reported that the modification of plant species composition is not the primary factor driving changes in nitrogen transformation processes in ecosystems experiencing grazing. It is likely that urine and dung deposition from livestock in grazed Phragmites plots stimulated an increase in N function genes, and the relevant N cycling processes were likely enhanced, which is consistent with results obtained from analysis of grazing on the alpine meadow [70]. However, these results conflict with those from the semi-arid grassland ecosystem [73]. The discrepancy in the grazing effect between wetland and grassland suggests that different soil properties and grazing intensities [74] may have variant effects on N cycling.

Most C fixation and degradation genes were significantly increased in samples from LWT sites (Figure 10 and Figure 11), suggesting that grazing may have effects on C pools and fluxes in degraded Phragmites wetlands. We noted that grazing inhibited the aboveground biomass; however, it accelerated C degrading processes, which was likely due to the enhanced labile portion of soil C and digested nutrient contents due to livestock manure deposition [70]. The results showed that the proportion of herbaceous vegetation was more abundant in restored Phragmites sites (Figure 2), which may have increased the recalcitrant C portion of the substrate input into the soil. Unsurprisingly, an analysis of RRs revealed that although the abundance of most genes involved in C degradation was unchanged compared with lightly degraded wetlands, those of ligninase were significantly increased at restored sites (*p* < 0.05; Figure S5a). Further, in the natural Carex sampling sites, the abundance of the C degrading genes *amyX*, *rgh*, and *mnp*, which are related to the degradation of starch, pectin utilization, and lignin decomposition, respectively, was significantly increased (Figure S5b). These results were similar to that observed in restored Phragmites sites. Our results of concurrent increases in the C degradation and N cycling processes in grazed wetland sites were consistent with findings from several grazed grassland studies [70,75,76]. However, they were inconsistent with those from several other studies conducted in an alpine meadow and semi-arid grassland ecosystems [46,77]. A possible explanation is that the increased heterogeneity of soil environments and different grazing intensities may have opposite effects on C/N pools and fluxes [70,74].

Four key functional genes related to methane cycling were detected as showing altered abundance. The abundance of the methanogenesis related genes *mcrA* and *hdrB*, and the *mmoX* gene for methane oxidation showed an increase in samples from restored Phragmites wetlands (*p* < 0.05; Figure 10c). Moreover, RRs showed that the abundance of *pmoA* was decreased in samples from severely degraded sites (*p* < 0.05). The abundance of these genes was positively correlated with methane production and consumption potential [78,79], indicating that the ecological processes of methane emission and uptake in the Phragmites soils were affected by wetland conditions. In combination with analysis of soil microbial community structure, the methanotrophic community, that was predominantly composed of *Methylococcales*, might facilitate methane oxidation in the restored Phragmites soils (Figure 3). In soil samples from Carex sites, the abundance of the *hdrB*, *pmoA*, and *mmoX* genes was related to microbial methane metabolism and was not significantly affected by wetland conditions, whereas *mcrA* gene abundance was significantly increased in samples from natural Carex sites, compared with declined sites (*p* < 0.05; Figure 10d). Accordingly, the methanogenic archaea (including *Methanomicrobiales*, *Methanocellales*, and *Methanobacteriales*) appeared to be sensitive to a decrease in *Carex* abundance as they were reduced at degraded wetland sites (Figure 4). Wetland soils are generally regarded as the largest global natural methane source and sink [80]. The area of wetland surface inundation, water table depth, soil moisture, and aquatic plants influence methanogenesis and methane consumption [81,82,83]. Notably, our results suggest that the decline in wetland vegetation at degraded sites decreases the production and uptake of methane in the soil and may cause the loss of the methane source and sink function, which strongly contributes to mitigating climate change [84]. In particular, human activities (e.g., grazing, wetland vegetation cover change) have modified these biogeochemical cycles and have likely further amplified ecosystem uncertainties in wetlands [19]. The results showed that most of the microbial functional genes associated with methane cycling were increased in restored Phragmites site samples, which may result from the increased soil carbon input by aboveground plants. However, unlike the methane functional genes, numerous C degrading genes were significantly increased in abundance in samples from grazing sites. While manure deposition from livestock in grazed plots could be partially attributed to increased C availability on surface soil, the stimulating effect of grazing on microbial genes involved in methanogenesis, the anaerobic process, might play a relatively minor role. The increase in below-ground litter inputs and root exudation is likely to be the dominant mechanism driving soil methane cycling in restored/natural wetland sites [85,86,87].

The results from Mantel tests in this study indicated that significant correlations exist between functional microbial community structure and vegetation coverage (*r* = 0.323, *p* < 0.05) and density (*r* = 0.628, *p* < 0.05), respectively, in samples from Phragmites sites. Therefore, changes in the aboveground vegetation could affect below-ground microbial assemblages [87]. Additionally, the explained variation in functional microbial potentials by the available soil variables was high. Soil pH, TN, and vegetation variables appeared to play an essential role in the variation of functional microbial community structure in samples from Phragmites wetlands, explaining 77.03% of the total variance. These results illustrate that changes in soil and vegetation properties of wetlands could affect soil microbial communities and their function, as they showed fundamentally different responses to degraded sites compared with restored sites (e.g., microbial carbon and nitrogen cycling routes in Figure S7). As shown in the conceptual diagram (Figure S7), microbial community composition and functional potentials are essential to the maintenance of carbon storage and regulation of greenhouse gas emissions in wetland soils.

### 4.3. Soil Microbial Community: An Important Indicator of Wetland Health or Restoration Status

Previous studies have shown that the composition of soil microbial communities can shift in response to changes in the wetland soil conditions [19,88]. Moreover, several studies have demonstrated that microbial groups are sensitive and non-resilient to disturbances immediately [89]; however, some researchers have shown that microbial communities can recover in a very short time in mixing aquatic ecosystems [90]. Generally, microbial communities appeared to be sensitive to a disturbed ecosystem, yet rarely few reports, by our knowledge, were related to the microbial community and their functions in response to restored ecosystems. Our results showed that the vegetation properties, soil WC, pH, and TN of lightly degraded sites were closer to those of the restored wetlands compared to the severely degraded Phragmites sites (Table 1). Considering the gradual environmental changes in these Phragmites sampling sites, we hypothesized that variations in microbial composition and activity would shift accordingly. Interestingly, we found that samples from degraded Phragmites wetlands were distinct from those from the restored wetlands, whereas the microbial community composition and functional structure in degraded sites tended to be increasingly similar to restored sites with improving Phragmites wetland gradients (Figure 7). It is likely that a change in the composition of the plant community and soil conditions led to a change in soil microbiota, and conversely, microbial communities and functions may have the capacity to indicate the trend for wetland ecosystems and changes in wetland biogeochemical cycling [19].

Meanwhile, interestingly, bacterial NMDS analysis revealed that samples in the first scaled dimension were more similar between restored Phragmites sites and natural Carex sites than any other paired comparison. Analogously, this also occurred in the microbial functional genes as per the NMDS analysis. Bacterial assemblages and functional structures were unexpectedly similar between healthy and restored wetland sites regardless of aboveground vegetation, which might reflect a more closely resembled invisible micro ecological balance in driving biogeochemical processes in these wetlands [91,92]. Actually, it is a common assumption that changes in the composition of the soil microbiome should be predictable from the aboveground plant [92]. However, the effects of the alteration of wetland environmental properties on the composition of aboveground vegetation may take years to become evident [93], whereas soil microorganisms could respond in a short time. For example, in the results, the degradation of Carex sites led to variations in microbial communities, which were found close to invasive *Artemisia* sites. It is difficult to directly measure the degradation or restoration status of *Carex* tussock due to the relatively long time taken by vegetation shifts to respond to environmental changes [15]. Moreover, environmental factors can have direct or indirect effects on soil microbial communities and vice versa [94]. Therefore, using microorganisms as ‘bio-indicators’ of wetland soil conditions or processes is an important step forward in understanding how wetland ecosystems may respond to ongoing environmental changes.

## 5. Conclusions

In this study, we demonstrated that microbial communities exhibit distinct compositions and/or functions in degraded sites and at restored/natural sites in two typical wetlands, the Phragmites and Carex marshes, respectively. The results from this study provide insight into the relationships between below-ground microbiota and the ecological traits of wetlands. Bacterial and fungal diversity at degraded sites was significantly decreased compared with restored/natural sites, indicating that soil microbial assemblages were sensitive to disturbances in the Phragmites and Carex marsh ecosystems. Importantly, the results showed that wetland microbial communities were able to indicate soil biogeochemical flux changes with land use alteration. Further, our findings have particular implications about how shifts in the composition of whole microbial communities could be a crucial ecological indicator reflecting changes in wetland status. By demonstrating the prediction of soil microbiota, this study signified wetland restoration was important in reconstructing the ecological balance and reversing wetland ecosystem degradation. Although, due to environmental heterogeneity and increasing climate uncertainty, it is unclear whether these conclusions can be generalized to other wetland ecosystem types, efforts to understand the responses of soil microbes and their potential impact are necessary to provide insight into below-ground wetland ecosystem functions.

## Figures and Tables

**Table 1:** Soil characteristics (mean ± standard error) of the degraded and restored Phragmites wetlands.

	Degraded Phragmites Wetlands			Restored Wetlands					
	LWT	L1	L2	B1	B2	W1	W2	WS1	WS2
pH	9.48 ± 0.29 a	8.67 ± 0.34 b	8.95 ± 0.24 ab	8.97 ± 0.20 ab	9.41 ± 0.19 a	8.83 ± 0.10 b	8.61 ± 0.32 b	7.98 ± 0.13 c	8.32 ± 0.35 bc
TN (g/kg)	1.51 ± 0.45 a	0.66 ± 0.16 bc	0.70 ± 0.08 b	0.81 ± 0.08 ab	0.41 ± 0.01 c	0.71 ± 0.05 b	1.14 ± 0.39 ab	0.80 ± 0.20 ab	0.62 ± 0.05 bc
TC (g/kg)	21.98 ± 3.77 ab	24.97 ± 5.22 a	25.00 ± 3.33 a	14.74 ± 1.10 b	10.39 ± 0.84 c	14.39 ± 1.14 b	18.39 ± 4.19 ab	15.50 ± 2.42 b	13.20 ± 0.22 bc
TP (g/kg)	0.34 ± 0.07 c	0.31 ± 0.05 c	0.20 ± 0.10 c	0.93 ± 0.11 a	0.47 ± 0.09 bc	0.50 ± 0.14 bc	0.60 ± 0.05 b	0.58 ± 0.10 b	0.60 ± 0.08 b
TS (g/kg)	7.93 ± 0.74 b	6.47 ± 2.05 b	11.07 ± 0.85 a	10.53 ± 2.70 ab	5.93 ± 1.10 b	8.13 ± 2.91 ab	8.03 ± 2.53 ab	6.33 ± 0.71 b	7.47 ± 1.25 b
WC (%)	15.52 ± 6.30 bc	19.11 ± 1.51 ab	19.33 ± 4.90 ab	6.50 ± 1.73 c	16.58 ± 2.24 b	29.40 ± 2.83 a	30.90 ± 8.20 a	28.59 ± 8.04 a	21.90 ± 1.64 a

- Different letters in the table indicate statistical differences at *p* < 0.05, calculated by one-way ANOVA.

**Table 2:** Soil characteristics (mean ± standard error) of the degraded and natural Carex wetlands.

	Degraded Carex Wetlands				Natural Carex Wetlands		
	H	C1	C2	C3	T1	T2	ZL
pH	7.20 ± 0.08 a	5.96 ± 0.25 c	6.38 ± 0.54 bc	6.74 ± 0.53 ab	6.55 ± 0.27 b	7.36 ± 0.32 a	5.49 ± 0.54 c
TN (g/kg)	1.37 ± 0.21 d	4.69 ± 1.08 a	2.27 ± 0.12 c	2.89 ± 0.16 b	1.07 ± 0.42 de	0.74 ± 0.31 e	2.25 ± 1.29 cd
TC (g/kg)	14.77 ± 1.82 d	47.24 ± 5.61 a	21.96 ± 0.97 c	26.78 ± 1.63 b	10.98 ± 4.32 d	8.64 ± 3.39 d	22.74 ± 10.58 bc
TP (g/kg)	0.51 ± 0.05 a	0.22 ± 0.02 b	0.41 ± 0.14 ab	0.47 ± 0.15 ab	0.49 ± 0.13 a	0.59 ± 0.08 a	0.38 ± 0.12 ab
TS (g/kg)	12.70 ± 2.14 ab	8.90 ± 1.60 b	11.67 ± 3.20 ab	11.43 ± 4.81 ab	14.33 ± 1.15 a	13.40 ± 2.36 ab	7.80 ± 2.41 b
WC (%)	10.71 ± 2.33 b	14.56 ± 6.56 b	12.13 ± 3.81 b	11.11 ± 3.44 b	34.68 ± 4.60 a	20.53 ± 6.26 b	50.23 ± 11.61 a

- Different letters in the table indicate statistical differences at *p* < 0.05, calculated by one-way ANOVA.

**Table 3:** Alpha-diversity, including observed-species, Shannon and Simpson indices of the soil bacterial and fungal community in the Phragmites marsh sampling sites.

**Bacteria α-diversity**					
	**LWT**	**L1**	**L2**	**B1**	**B2**
OS	2732.00 ± 121.87 b	3345.00 ± 194.01 a	3350.00 ± 373.37 a	3467.67 ± 214.95 a	3570.67 ± 131.70 a
Shannon	9.107 ± 0.090 c	9.486 ± 0.296 bc	9.427 ± 0.336 bc	9.593 ± 0.228 b	9.869 ± 0.115 ab
Simpson	0.994 ± 0.001 b	0.990 ± 0.008 b	0.991 ± 0.002 b	0.993 ± 0.002 b	0.996 ± 0.000 a
	**W1**	**W2**	**WS1**	**WS2**	
OS	3498.00 ± 258.20 a	3437.67 ± 619.30 a	3863.33 ± 262.95 a	3783.33 ± 110.82 a	
Shannon	9.607 ± 0.156 b	9.260 ± 1.543 bc	10.053 ± 0.151 a	10.017 ± 0.050 a	
Simpson	0.993 ± 0.002 b	0.976 ± 0.037 b	0.997 ± 0.001 a	0.997 ± 0.001 a	
**Fungi α-diversity**					
	**LWT**	**L1**	**L2**	**B1**	**B2**
OS	690.33 ± 103.88 d	1363.00 ± 195.64 bc	1156.67 ± 126.59 c	1318.00 ± 221.03 bc	1118.00 ± 293.25 c
Shannon	4.206 ± 0.503 c	5.762 ± 0.251 b	5.428 ± 0.715 bc	4.597 ± 1.071 bc	4.273 ± 0.446 c
Simpson	0.829 ± 0.078 b	0.920 ± 0.033 a	0.885 ± 0.083 ab	0.858 ± 0.101 ab	0.849 ± 0.049 b
	**W1**	**W2**	**WS1**	**WS2**	
OS	1496.00 ± 109.38 b	1655.00 ± 263.61 ab	1952.67 ± 137.42 a	1145.33 ± 113.60 c	
Shannon	4.931 ± 0.966 bc	6.425 ± 0.601 ab	6.465 ± 0.303 a	4.515 ± 0.202 c	
Simpson	0.835 ± 0.105 b	0.962 ± 0.014 a	0.940 ± 0.026 a	0.851 ± 0.023 b	

- OS: observed-species indices. - Different letters in the table indicate statistical differences at *p* < 0.05, calculated by one-way ANOVA.

**Table 4:** Alpha-diversity, including observed-species, Shannon and Simpson indices of the soil bacterial and fungal community in the Carex marsh sampling sites.

**Bacteria α-diversity**				
	**H**	**C1**	**C2**	**C3**
OS	3243.67 ± 171.56 b	3281.33 ± 142.98 b	3093.00 ± 47.62 b	3291.33 ± 188.25 b
Shannon	9.846 ± 0.184 b	9.827 ± 0.097 b	9.789 ± 0.060 b	9.806 ± 0.132 b
Simpson	0.997 ± 0.001 a	0.997 ± 0.000 a	0.997 ± 0.000 a	0.997 ± 0.001 a
	**T1**	**T2**	**ZL**	
OS	4167.33 ± 155.55 a	4105.67 ± 218.93 a	3833.67 ± 227.53 a	
Shannon	10.193 ± 0.130 ab	10.270 ± 0.100 a	10.008 ± 0.206 ab	
Simpson	0.997 ± 0.001 a	0.997 ± 0.001 a	0.996 ± 0.001 a	
**Fungi α-diversity**				
	**H**	**C1**	**C2**	**C3**
OS	926.67 ± 107.29 b	578.33 ± 23.12 c	634.67 ± 38.28 c	777.67 ± 130.45 bc
Shannon	5.526 ± 0.408 a	4.840 ± 0.105 b	5.218 ± 0.139 a	5.284 ± 0.679 a
Simpson	0.928 ± 0.024 a	0.918 ± 0.014 a	0.937 ± 0.008 a	0.921 ± 0.066 a
	**T1**	**T2**	**ZL**	
OS	1875.00 ± 316.70 a	1528.67 ± 242.08 a	2088.67 ± 325.77 a	
Shannon	4.993 ± 0.815 ab	5.949 ± 0.125 a	6.537 ± 1.026 a	
Simpson	0.843 ± 0.086 a	0.953 ± 0.018 a	0.940 ± 0.048 a	

- OS: observed-species indices. - Different letters in the table indicate statistical differences at *p* < 0.05, calculated by one-way ANOVA.

## Data Availability

The data presented in this study are openly available in the NCBI Sequence Read Archive, the accession number PRJNA660301 and PRJNA660325.

## Supplementary Materials

The following are available online at https://www.mdpi.com/2076-2607/9/2/211/s1. Figure S1: The relative abundance in dominant phyla of the soil bacterial (a) and fungal (b) community in the Phragmites and the Carex marsh sampling sites. The relative abundance lower than 1% were assigned as “Others”. Figure S2: Comparisons of the dissimilarity of functional community structure via the ANOSIM tests based on the Bray-Curtis dissimilarity index in Phragmites (a) and Carex (b) sites, respectively. Figure S3: The response of N cycling-related genes to degraded and restored/natural wetland sites as per GeoChip data. (a,b) Normalized GeoChip signal intensities of genes involved in the N cycling at restored (W), lightly degraded (L), and severely degraded (LWT) Phragmites marsh sites, and the natural (T, ZL) and degraded (C) Carex marsh sites, respectively. Error bars represent one standard deviation of the mean (*n* = 3). ***, *p* < 0.001, **, *p* < 0.01, *, *p* < 0.05, based on unpaired *t*-tests. Figure S4: Response of C fixation and methane metabolism-related genes to degraded and restored/natural wetland sites according to GeoChip data. (a,b) Normalized GeoChip signal intensities of genes involved in C fixation and methane metabolism in the restored (W), lightly degraded (L), and severely degraded (LWT) Phragmites marsh sites, and the natural (T, ZL) and degraded (C) Carex marsh sites, respectively. Error bars represent one standard deviation of the mean (*n* = 3). ***, *p* < 0.001, **, *p* < 0.01, *, *p* < 0.05, based on unpaired *t*-test. Figure S5: RRs of C decomposition related genes (a) are presented as the difference between the lightly degraded and severely degraded Phragmites marsh sites (red symbols), and between the restored and lightly degraded Phragmites marsh sites (blue symbols); (b) the difference between the natural and degraded Carex marsh sites (red symbols), and between two natural Carex marsh sites (blue symbols). The RR is considered significant when the 95% confidence interval (presented as error bars) does not overlap with 0. Figure S6: CCA and VPA analysis based on selected environmental variables and 16S rRNA gene amplicon sequencing of all samples in Phragmites (a,b) and Carex (c,d) sites, or ITS gene amplicon sequencing of all samples in Phragmites (e,f) and Carex (g,h) sites. The percentage values of axis 1 and 2 in CCA indicates the percentage of variation explained by the corresponding axes. Environmental variables in VPA are divided into groups of soil and plant properties. The variance unexplained by the tested variable groups is indicated in the VPA figure. Figure S7: Conceptual diagram of functional microbial responses and the effects of degraded sites on wetland ecosystems (a) and restored sites (b) in the Phragmites marsh. Substrate pools are shown in yellow rectangles, gases in blue rectangles, biological processes of C/N cycle in pink parallelograms, enhanced biological processes of C/N cycle in red parallelograms, plant processes in green parallelograms, and the grazing effect in gray parallelograms. Material flows are indicated by black arrows. The impact of microbial effects are marked by red arrows, and labeled with a ‘+’ for positive effect, and ‘-’ for negative effect. Table S1: Characteristics and description of sampling sites in the Phragmites and Carex marshes. Table S2: Pairwise comparisons of the structure of soil bacterial communities between samples from the Phragmites or Carex sites, respectively. Table S3: Pairwise comparisons for the structure of soil fungal communities between samples from the Phragmites or Carex sites, respectively. Table S4: Correlation between the bacterial/fungal communities and environmental variables in the Phragmites sites as shown by Mantel tests. Table S5: Correlation between the bacterial/fungal communities and environmental variables in the Carex sites as shown by Mantel tests. Table S6: Correlation between all detected N and C cycling related functional genes in microbial communities and environmental variables in samples from the Phragmites and Carex sites as shown by Mantel tests. Table S7: Correlation between all detected N and C cycling-related functional genes in microbial communities and the environmental variables in samples from the *Phragmites* or *Carex* sites respectively, as shown by Mantel tests.
