# Supplementary material for: Disentangling Responses of the Subsurface Microbiome to Wetland Status and Implications for Indicating Ecosystem Functions
Source: Microorganisms. 2021 Jan 20;9(2):211. doi: 10.3390/microorganisms9020211 (PMC7909544; doi:10.3390/microorganisms9020211)
Supplement: Supplementary file 1 [file microorganisms-09-00211-s001.pdf]

Disentangling responses of the subsurface microbiome to wetland status and implications for indicating ecosystem functions

Jie Gao[1,2], Miao Liu[3], Sixue Shi[2,3], Ying Liu[1], Yu Duan[4], Xianguo Lv[5], Tsing Bohu[6],Yuehui Li[3], Yuanman Hu[3], Na Wang[1,2], Qiuying Wang[1,2], Guoqiang Zhuang[1,2*], Xuliang Zhuang[1,2*]

[1] CAS Key Laboratory of Environmental Biotechnology, Research Center for Eco-Environmental Sciences, Chinese Academy of Sciences, Beijing 100085, China;
[2] College of Resources and Environment, University of Chinese Academy of sciences, Beijing 100049, China;
[3] CAS Key Laboratory of Forest Ecology and Management, Institute of Applied Ecology, Chinese Academy of Sciences, Shenyang 110016, China;
[4] Beijing Enterprises Water Group Limited, Beijing 100124, China;
[5] Key Laboratory of Wetland Ecology and Environment, Northeast Institute of Geography and Agroecology, Chinese Academy of Sciences, Changchun, Jilin 130102, China；
[6] CSIRO Mineral Resources, 26 Dick Perry Avenue, Kensington, Western Australia 6151, Australia
* Correspondence: gqzhuang@rcees.ac.cn; Tel: +86-10-62849613;
xlzhuang@rcees.ac.cn; Tel.: +86-10-62849193

Figures

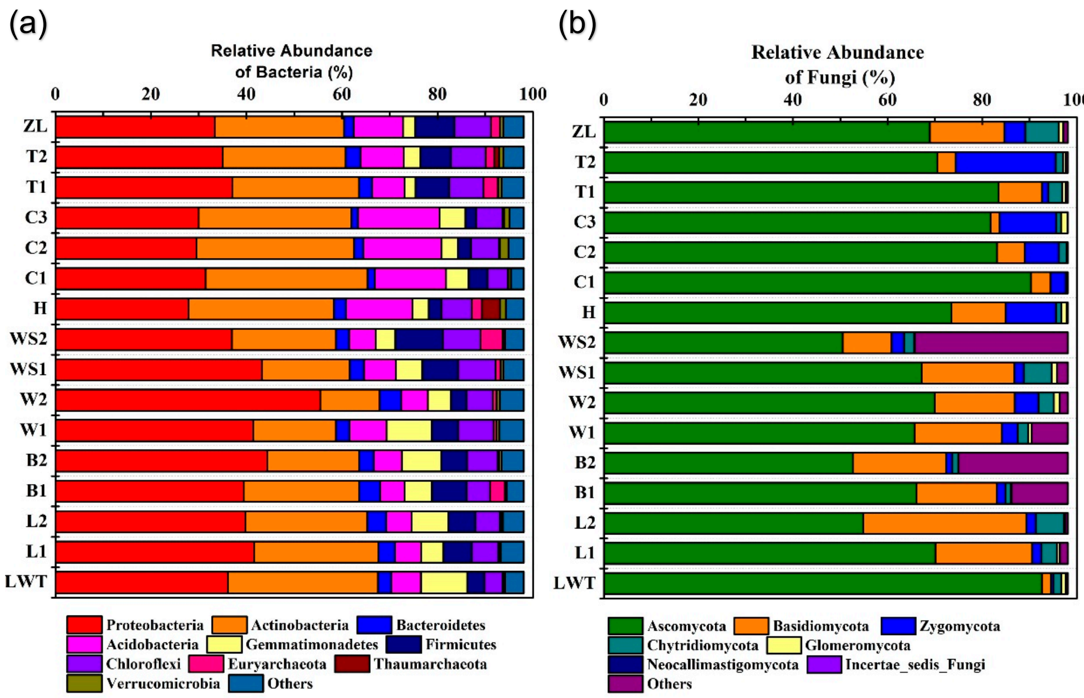

Fig S1. The relative abundance in dominant phyla of the soil bacterial (a) and fungal (b) community in the Phragmites and the Carex marsh sampling sites. The relative abundance lower than 1% were assigned as “Others”.

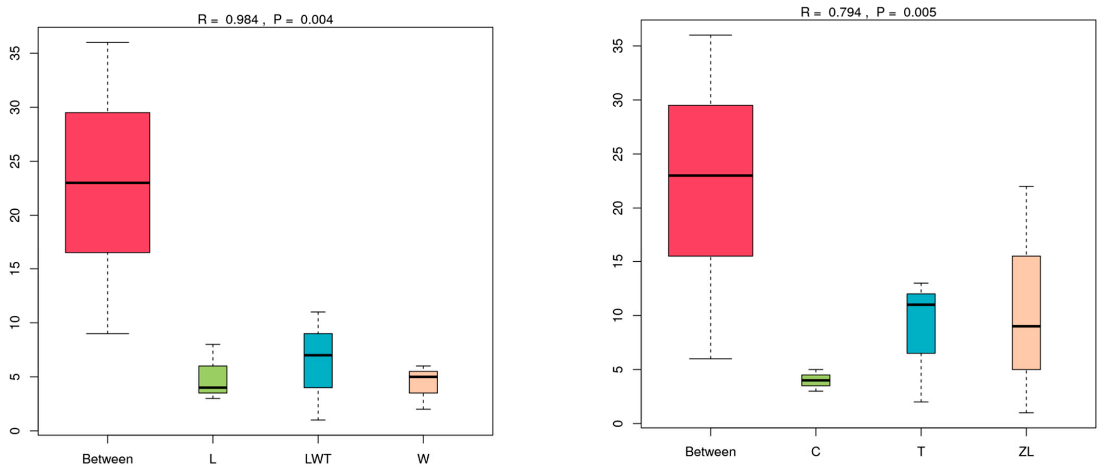

Fig S2. Comparisons of the dissimilarity of functional community structure via the ANOSIM tests based on the Bray-Curtis dissimilarity index in *Phragmites* (a) and *Carex* (b) sites, respectively.

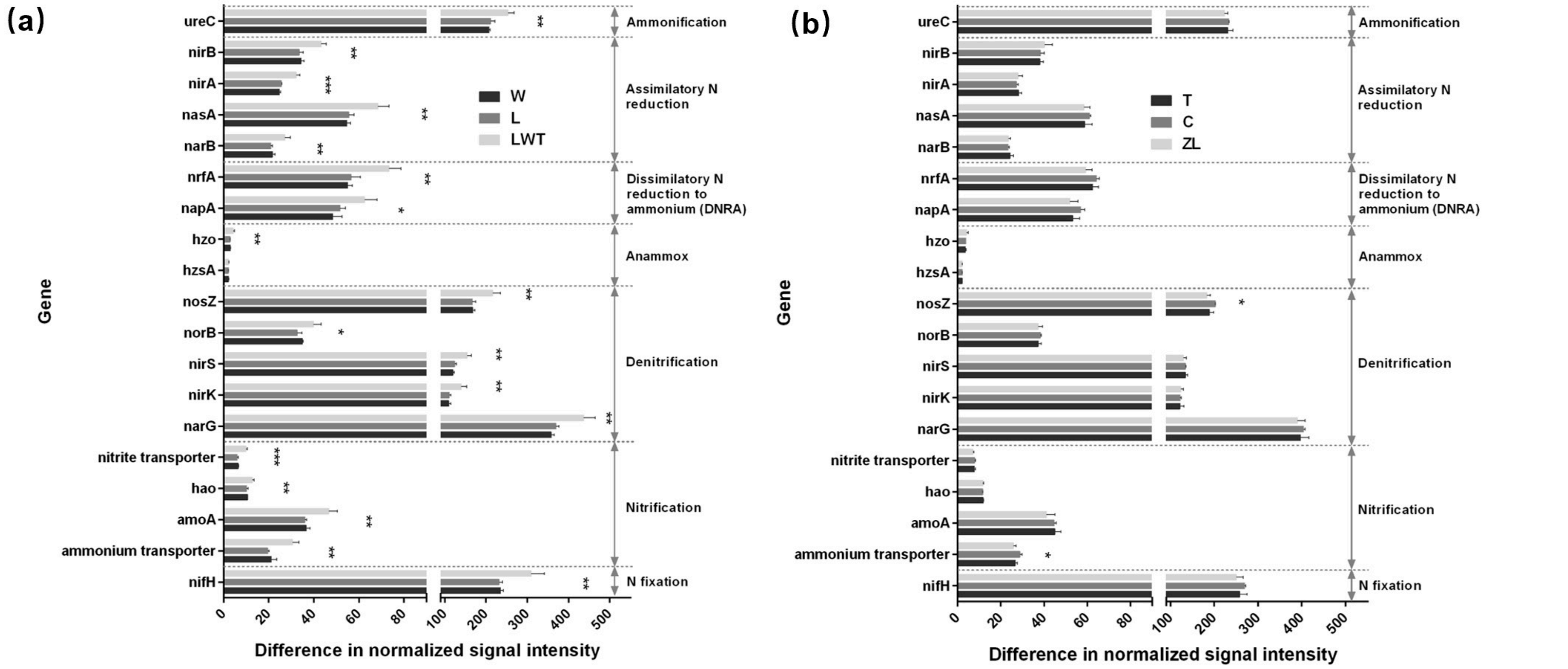

Fig S3. The response of N cycling-related genes to degraded and restored/natural wetland sites as per GeoChip data. (a-b) Normalized GeoChip signal intensities of genes involved in the N cycling at restored (W), lightly degraded (L), and severely degraded (LWT) Phragmites marsh sites, and the natural (T, ZL) and degraded (C) Carex marsh sites, respectively. Error bars represent one standard deviation of the mean ($n = 3$). ***, $p < 0.001$, **, $p < 0.01$, *, $p < 0.05$, based on unpaired t-tests.

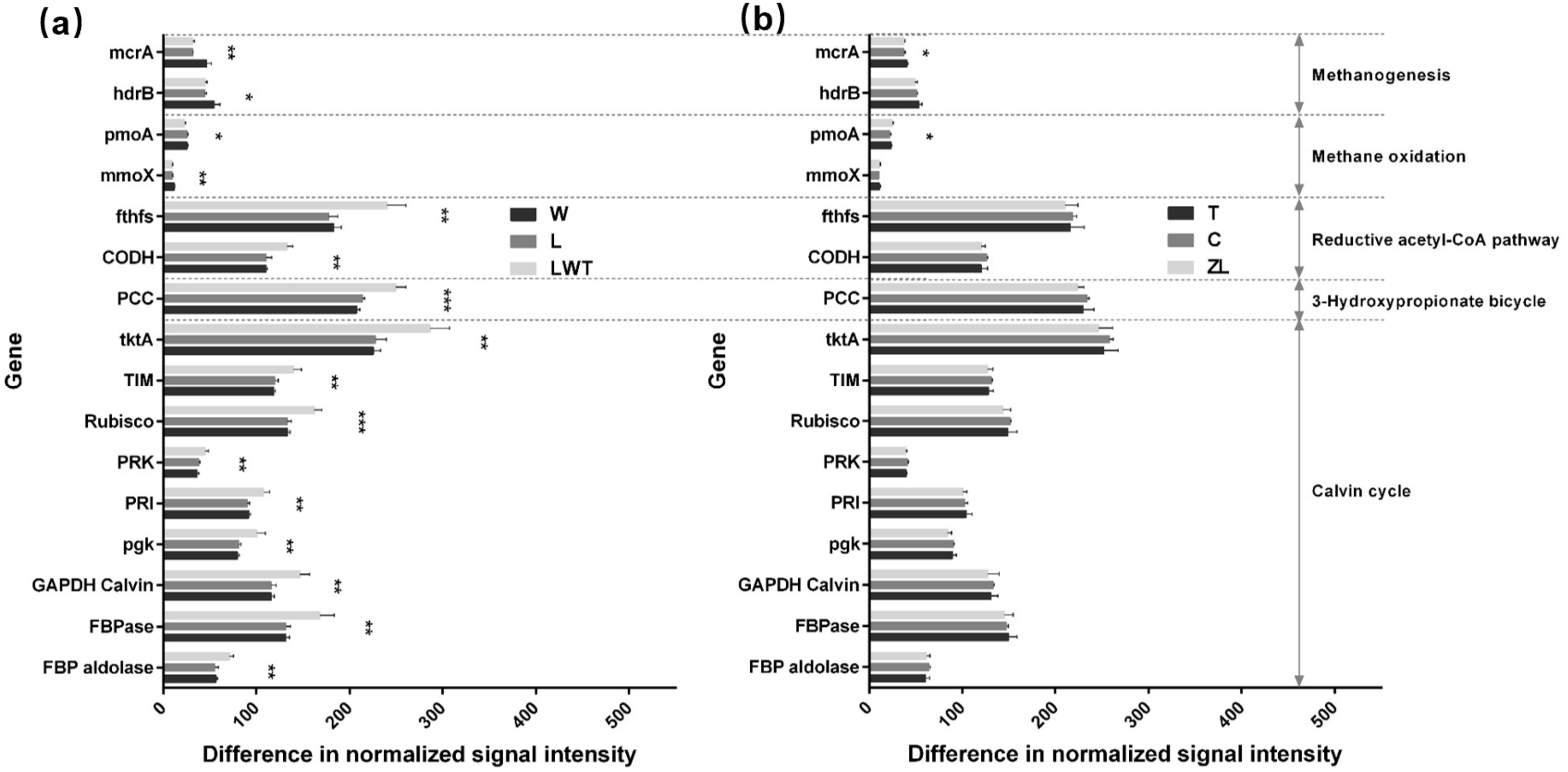

Fig S4. Response of C fixation and methane metabolism-related genes to degraded and restored/natural wetland sites according to GeoChip data. (a-b) Normalized GeoChip signal intensities of genes involved in C fixation and methane metabolism in the restored (W), lightly degraded (L), and severely degraded (LWT) Phragmites marsh sites, and the natural (T, ZL) and degraded (C) Carex marsh sites, respectively. Error bars represent one standard deviation of the mean ($n = 3$). ***, $p < 0.001$, **, $p < 0.01$, *, $p < 0.05$, based on unpaired t-test.

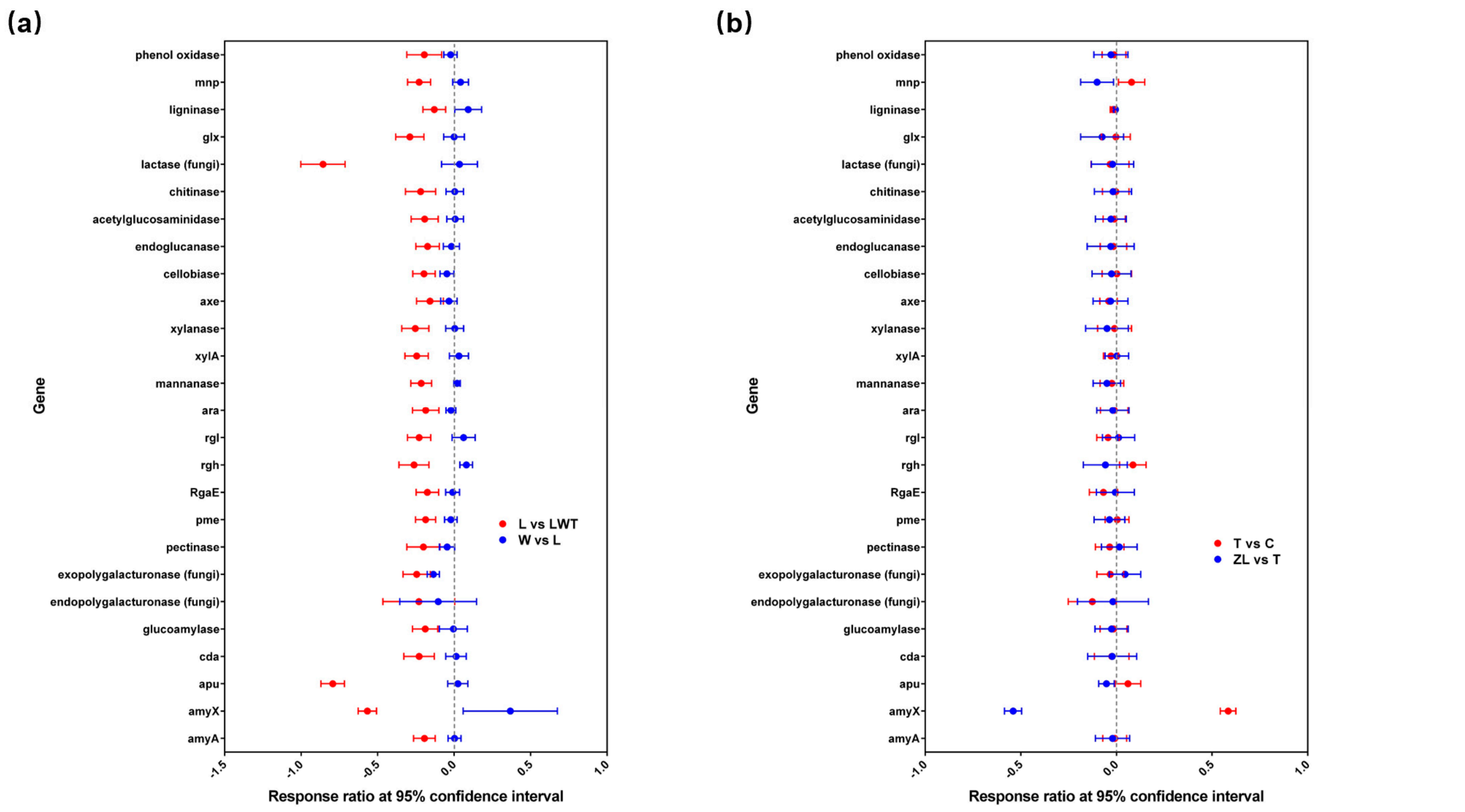

Fig S5. RRs of C decomposition related genes (a) are presented as the difference between the lightly degraded and severely degraded *Phragmites* marsh sites (red symbols), and between the restored and lightly degraded *Phragmites* marsh sites (blue symbols); (b) the difference between the

natural and degraded *Carex* marsh sites (red symbols), and between two natural *Carex* marsh sites (blue symbols). The RR is considered significant when the 95% confidence interval (presented as error bars) does not overlap with 0.

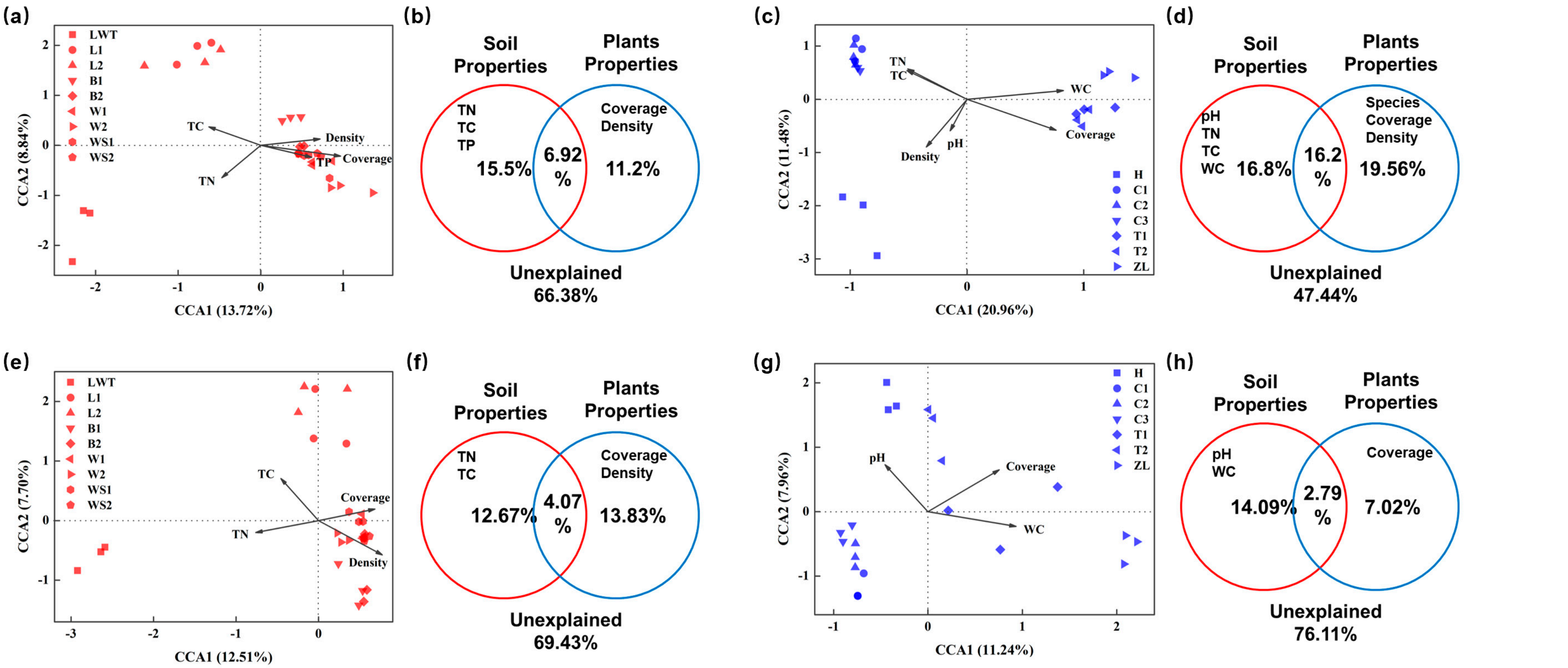

Fig S6. CCA and VPA analysis based on selected environmental variables and 16S rRNA gene amplicon sequencing of all samples in *Phragmites*

(a-b) and *Carex* (c-d) sites, or ITS gene amplicon sequencing of all samples in *Phragmites* (e-f) and *Carex* (g-h) sites. The percentage values of axis 1 and 2 in CCA indicates the percentage of variation explained by the corresponding axes. Environmental variables in VPA are divided into groups of soil and plant properties. The variance unexplained by the tested variable groups is indicated in the VPA figure.

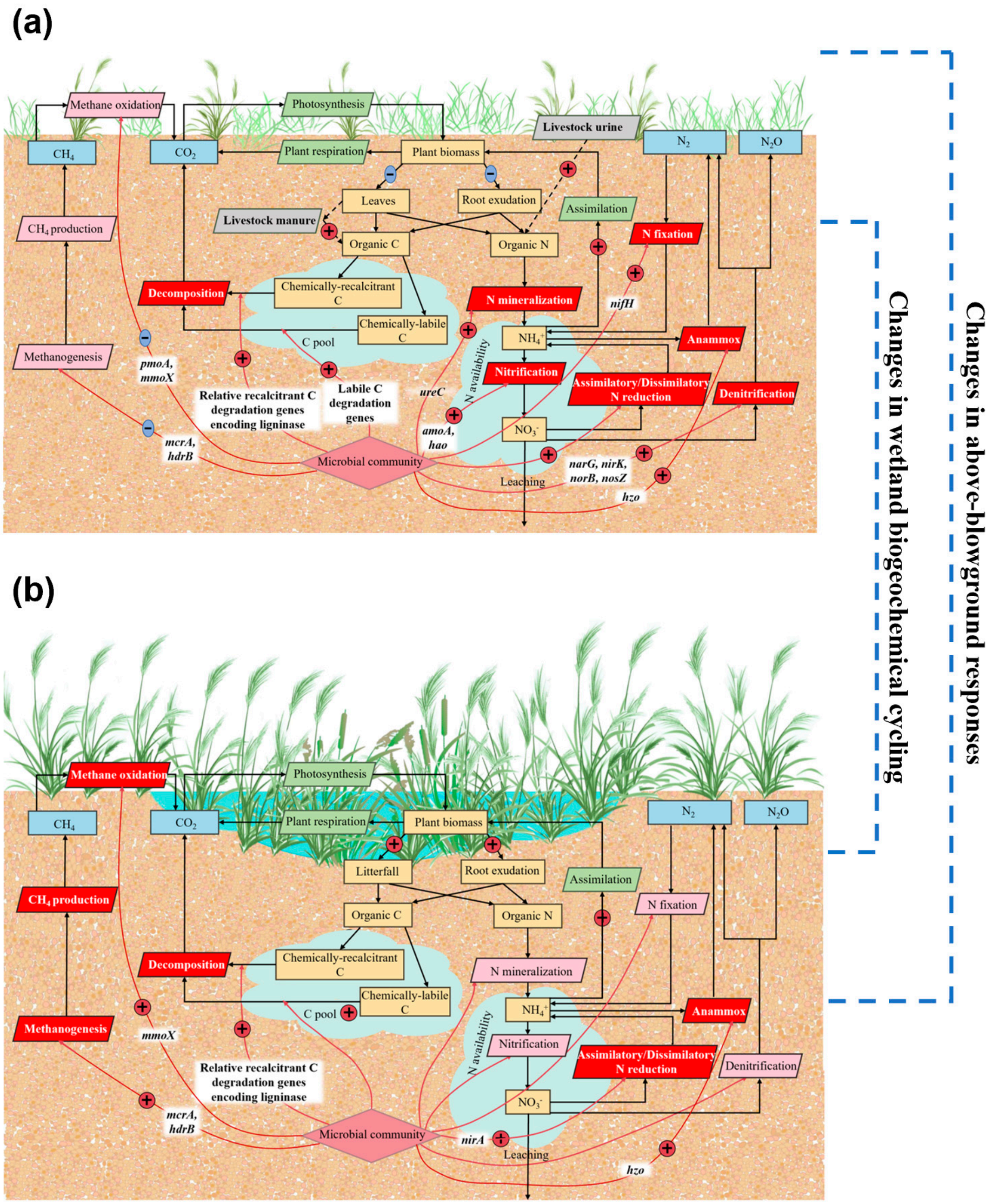

Fig S7. Conceptual diagram of functional microbial responses and the effects of degraded sites on wetland ecosystems (a) and restored sites (b) in the Phragmites marsh. Substrate pools are shown in yellow rectangles, gases in blue rectangles, biological processes of C/N cycle in pink parallelograms, enhanced biological processes of C/N cycle in red parallelograms, plant processes in green parallelograms, and the grazing effect in gray parallelograms. Material flows are indicated by black arrows. The impact of microbial effects are marked by red arrows, and labeled with a '+' for positive effect, and '-' for negative effect.

Tables

Table S1 Characteristics and description of sampling sites in the Phragmites and Carex marshes

Phragmites marsh						
Sample name	Major species	Sample description	Sampling sites	Location	Sampling site description	GeoChip analysis
LWT	*Phragmites australis; Leymus chinensis*	*P. australis* rhizosphere soil (0-15 cm)	123°11'0.55" 46°9'3.98"		Degraded wetland: *Phragmites* and *Leymus chinensis* meadow, grazing territories, outside Momoge National Nature Reserve	Yes, named LWT in GeoChip analysis
L1	*Phragmites australis*	*P. australis* rhizosphere soil (0-15 cm)	123°36'7.81" 45°55'18.33"		Degraded wetland: Phragmites marsh of low soil moisture, in Momoge National Nature Reserve	-
L2	*Phragmites australis*	*P. australis* rhizosphere soil (0-15 cm)	123°36'10.69" 45°55'16.39"		Degraded wetland: Phragmites marsh of low soil moisture, in Momoge National Nature Reserve	Yes, named L in GeoChip analysis
B1	*Scirpus planiculmis*	*S. planiculmis* rhizosphere soil (0-15 cm)	123°37'21.80" 45°53'40.35"	Momoge wetland	Restored wetland: *S. planiculmis* vegetation restoration in Momoge National Nature Reserve	-
B2	*Scirpus planiculmis*	*S. planiculmis* rhizosphere soil (0-15 cm)	123°37'19.57" 45°53'39.85"		Restored wetland: *S. planiculmis* vegetation restoration in Momoge National Nature Reserve	-
W1	*Phragmites australis*	*P. australis* rhizosphere soil (0-15 cm)	123°37'23.59" 45°53'41.37"		Restored wetland: *P. australis* vegetation restoration in Momoge National Nature Reserve	Yes, named W in GeoChip analysis
W2	*Phragmites australis*	*P. australis* rhizosphere soil (0-15 cm)	123°37'19.26" 45°53'41.18"		Restored wetland: *P. australis* vegetation restoration in Momoge National Nature Reserve	-

WS1	*Phragmites australis; Typha angustifolia*	*P. australis* rhizosphere soil (0-15 cm)	123°37'22.66" 45°53'42.11"			Restored wetland: *P. australis* vegetation restoration in Momoge National Nature Reserve, nearer to lake	-
WS2	*Phragmites australis; Typha angustifolia*	*P. australis* rhizosphere soil (0-15 cm)	123°37'20.33" 45°53'41.79"			Restored wetland: *P. australis* vegetation restoration in Momoge National Nature Reserve, nearer to lake	-

Carex marsh

Sample name	Major species	Sample description	Sampling sites		Location	Sampling site description	GeoChip analysis
H	*Artemisia* sp.	*Artemisia* sp. rhizosphere soil (0-15 cm)	123°58'30.87" 45°52'19.71"			Degraded wetland: mesophytes invasion in *Carex* sp. marsh	-
C1	*Carex* sp.; *Deyeuxia purpurea*	*Carex* sp. rhizosphere soil (0-15 cm)	123°58'20.03" 45°52'18.78"			Degraded wetland: boundaries of *Artemisia* sp. and *Carex* sp. marsh	-
C2	*Carex* sp.; *Deyeuxia purpurea*	*Carex* sp. rhizosphere soil (0-15 cm)	123°58'15.29" 45°52'15.79"			Degraded wetland: near the boundaries of *Artemisia* sp. and *Carex* sp. marsh	-
C3	*Carex* sp.; *Deyeuxia purpurea*	*Carex* sp. rhizosphere soil (0-15 cm)	123°58'6.38"	45°52'16.11"	Momoge wetland	Degraded wetland: far from the boundaries of *Artemisia* sp. and *Carex* sp. marsh	Yes, named C in GeoChip analysis
T1	*Carex* sp.; *Polygonum persicaria; Deyeuxia purpurea*	*Carex* sp. rhizosphere soil (0-15 cm)	123°56'52.68"	45°54'0.47"		Natural wetland: less human disturbance, natural *Carex* tussock marsh	Yes, named T in GeoChip analysis
T2	*Carex* sp.; *Polygonum persicaria; Deyeuxia purpurea*	*Carex* sp. rhizosphere soil (0-15 cm)	123°56'53.88"	45°54'0.57"		Natural wetland: less human disturbance, natural *Carex* tussock marsh	-
ZL	*Carex* sp.; *Deyeuxia purpurea*	*Carex* sp. rhizosphere soil (0-15 cm)	130°56'30.30" 47°18'45.99"		Duluhe wetland	Natural wetland: less human disturbance, natural *Carex* tussock marsh	Yes, named ZL in GeoChip analysis

Table S2 Pairwise comparisons of the structure of soil bacterial communities between samples from the *Phragmites* or *Carex* sites, respectively.

Sites compared	ANOSIM.*r*	ANOSIM.*P*	PERMANOVA.*F*	PERMANOVA.*P*
Phragmites vs. *Carex*	**0.8714**	**0.001*****	**18.8986**	**0.001*****
LWT vs. L	**0.9876**	**0.016***	**6.0199**	**0.008****
LWT vs. W	**1**	**0.009****	**7.7876**	**0.013***
LWT vs. WS	**1**	**0.016***	**10.5056**	**0.012***
L vs. W	**0.9259**	**0.005****	**6.2024**	**0.003****
L vs. WS	**0.9277**	**0.003****	**6.9318**	**0.004****
W vs. B	**0.4277**	**0.004****	**3.2242**	**0.003****
W vs. WS	**0.5166**	**0.003****	**3.5652**	**0.004****
WS vs. B	**0.7259**	**0.002****	**4.5145**	**0.002****
L1 vs. L2	0.1481	0.2	1.1094	0.2
W1 vs. W2	0.3703	0.1	2.3342	0.1
WS1 vs. WS2	0.6666	0.1	2.6623	0.1
B1 vs. B2	0.6666	0.1	2.8887	0.1
H vs. C	**0.9772**	**0.003****	**5.9034**	**0.003****
H vs. T	**1**	**0.016***	**7.5614**	**0.015***
C vs. T	**1**	**0.001*****	**16.3781**	**0.001*****
C vs. ZL	**1**	**0.004****	**11.4655**	**0.006****
T vs. ZL	**1**	**0.015***	**5.3721**	**0.012***
C1 vs. C2	0.8518	0.1	2.9868	0.1
C2 vs. C3	0.8148	0.1	2.0085	0.1
C1 vs. C3	0.8518	0.1	3.0097	0.1
T1 vs. T2	0.8518	0.1	1.5939	0.1

***, $p < 0.001$, **, $p < 0.01$, *, $p < 0.05$, based on ANOSIM and PERMANOVA tests

Table S3 Pairwise comparisons for the structure of soil fungal communities between samples from the *Phragmites* or *Carex* sites, respectively.

Sites compared	ANOSIM.*r*	ANOSIM.*P*	PERMANOVA.*F*	PERMANOVA.*P*
Phragmites vs. *Carex*	**0.7815**	**0.001*****	**9.7394**	**0.001*****
LWT vs. L	**0.9938**	**0.016***	**4.3738**	**0.008****
LWT vs. W	**1**	**0.017***	**8.4609**	**0.016***
LWT vs. WS	**1**	**0.013***	**8.4609**	**0.011***
L vs. W	**0.4981**	**0.001*****	**2.6986**	**0.001*****
L vs. WS	**0.5666**	**0.002****	**3.6428**	**0.006****
W vs. B	**0.7462**	**0.002****	**4.6568**	**0.004****
W vs. WS	0.2333	0.079	**2.2322**	**0.041***
WS vs. B	**0.6407**	**0.004****	**4.6986**	**0.006****
L1 vs. L2	-0.1851	0.9	0.8226	0.8
W1 vs. W2	0.9259	0.1	3.2396	0.1
WS1 vs. WS2	0.9259	0.1	5.9035	0.1
B1 vs. B2	-0.1111	0.8	0.8915	0.5
H vs. C	**0.6144**	**0.003****	**3.0914**	**0.013***
H vs. T	**0.3456**	**0.044***	**2.1407**	**0.008****
C vs. T	**0.7109**	**0.001*****	**4.1152**	**0.001*****
C vs. ZL	**1**	**0.005****	**5.327**	**0.003****
T vs. ZL	**0.358**	**0.046***	**2.0359**	**0.029***
C1 vs. C2	1	0.1	8.7447	0.1
C2 vs. C3	1	0.1	10.0435	0.1
C1 vs. C3	1	0.1	11.4942	0.1
T1 vs. T2	0.7777	0.1	2.3311	0.1

***, $p < 0.001$, **, $p < 0.01$, *, $p < 0.05$, based on ANOSIM and PERMANOVA tests

Table S4 Correlation between the bacterial/fungal communities and environmental variables in the *Phragmites* sites as shown by Mantel tests.

	Bacteria		Fungi	
	r	*p*	*r*	*p*
Envs				
pH	0.0513	0.3	0.1095	0.128
TN	**0.2649**	**0.028****	**0.3984**	**0.001*****
TC	**0.2632**	**0.013***	**0.3645**	**0.001*****
TP	**0.2531**	**0.011***	0.1552	0.073
TS	0.0513	0.296	0.0277	0.319
WC	0.1082	0.147	0.0706	0.217
Veg				
Species	-0.0777	0.745	0.0997	0.141
Coverage	**0.6369**	**0.001*****	**0.6053**	**0.001*****
Density	**0.3481**	**0.002****	**0.4185**	**0.001*****

***, $p < 0.001$, **, $p < 0.01$, *, $p < 0.05$

Table S5 Correlation between the bacterial/fungal communities and environmental variables in the *Carex* sites as shown by Mantel tests.

	Bacteria		Fungi	
	r	*p*	*r*	*p*
Envs				
pH	**0.15**	**0.037***	**0.2059**	**0.019***
TN	**0.2231**	**0.011***	0.1217	0.118
TC	**0.188**	**0.02***	0.1464	0.075
TP	0.0355	0.263	0.0892	0.128
TS	0.052	0.212	0.0466	0.242
WC	**0.4962**	**0.001*****	**0.4813**	**0.001*****
Veg				
Species	**0.2699**	**0.002****	0.1176	0.15
Coverage	**0.5323**	**0.001*****	**0.3606**	**0.002****
Density	**0.2572**	**0.006****	0.1084	0.156

***, $p < 0.001$, **, $p < 0.01$, *, $p < 0.05$

Table S6 Correlation between all detected N and C cycling related functional genes in microbial communities and environmental variables in samples from the *Phragmites* and *Carex* sites as shown by Mantel tests.

	r	*p*
Envs		
pH	**0.31**	**0.001*****
TN	0.0065	0.429
TC	-0.0177	0.506
TP	-0.06	0.671
TS	-0.0711	0.754
WC	-0.0839	0.697
Veg		
Species	**0.3104**	**0.001*****
Coverage	**0.4191**	**0.001*****
Density	**0.5165**	**0.001*****

***, $p < 0.001$, **, $p < 0.01$, *, $p < 0.05$

Table S7 Correlation between all detected N and C cycling-related functional genes in microbial communities and the environmental variables in samples from the *Phragmites* or *Carex* sites respectively, as shown by Mantel tests.

	Phragmites		*Carex*	
	r	*p*	*r*	*p*
Envs				
pH	**0.5422**	**0.008****	0.1727	0.162
TN	**0.5933**	**0.007****	-0.0154	0.481
TC	0.0865	0.252	-0.0105	0.509
TP	-0.0247	0.474	0.0893	0.301
TS	-0.2059	0.925	0.2351	0.118
WC	0.0247	0.401	**0.3476**	**0.02***
Veg				
Coverage	**0.323**	**0.049***	**0.4479**	**0.007****
Density	**0.628**	**0.012***	0.0638	0.345

***, $p < 0.001$, **, $p < 0.01$, *, $p < 0.05$
